# *Agrobacterium* VirE2 Protein Modulates Plant Gene Expression and Mediates Transformation From Its Location Outside the Nucleus

**DOI:** 10.3389/fpls.2021.684192

**Published:** 2021-06-04

**Authors:** Rachelle A. Lapham, Lan-Ying Lee, Eder Xhako, Esteban Gañán Gómez, V. M. Nivya, Stanton B. Gelvin

**Affiliations:** ^1^Department of Biological Sciences, Purdue University, West Lafayette, IN, United States; ^2^Departamento de Ciencias Biológicas, Universidad EAFIT, Medellín, Colombia; ^3^Department of Plant Science, School of Biological Science, Central University of Kerala, Kasaragod, India

**Keywords:** *Agrobacterium*, *Arabidopsis*, plant transformation, VirE2, protein subcellular localization, transcriptome, proteome

## Abstract

*Agrobacterium* effector protein VirE2 is important for plant transformation. VirE2 likely coats transferred DNA (T-DNA) in the plant cell and protects it from degradation. VirE2 localizes to the plant cytoplasm and interacts with several host proteins. Plant-expressed VirE2 can complement a *virE2* mutant *Agrobacterium* strain to support transformation. We investigated whether VirE2 could facilitate transformation from a nuclear location by affixing to it a strong nuclear localization signal (NLS) sequence. Only cytoplasmic-, but not nuclear-localized, VirE2 could stimulate transformation. To investigate the ways VirE2 supports transformation, we generated transgenic *Arabidopsis* plants containing a *virE2* gene under the control of an inducible promoter and performed RNA-seq and proteomic analyses before and after induction. Some differentially expressed plant genes were previously known to facilitate transformation. Knockout mutant lines of some other VirE2 differentially expressed genes showed altered transformation phenotypes. Levels of some proteins known to be important for transformation increased in response to VirE2 induction, but prior to or without induction of their corresponding mRNAs. Overexpression of some other genes whose proteins increased after VirE2 induction resulted in increased transformation susceptibility. We conclude that cytoplasmically localized VirE2 modulates both plant RNA and protein levels to facilitate transformation.

## Introduction

*Agrobacterium tumefaciens*, the causative agent of crown gall disease, transfers virulence effector proteins to infected host plants to facilitate the transfer of T-(transfer) DNA into and trafficking through plant cells. Once in the nucleus, transferred DNA (T-DNA) uses the host’s machinery to express transgenes, and may integrate into the host genome. Scientists have used this process to insert beneficial genes into plants by replacing native T-DNA genes with other genes of interest, making *Agrobacterium*-mediated transformation the preferred method for plant genetic engineering (Gelvin, 2003, 2012; Pitzschke and Hirt, 2010; Lacroix and Citovsky, 2013, 2019; Hiei et al., 2014; Nester, 2015; Van Eck, 2018).

VirE2 is one of the *A. tumefaciens* effector proteins that is important for plant transformation (Gelvin, 2003, 2012). *A. tumefaciens* mutant strains lacking a functional *virE2* gene are severely attenuated in virulence (Stachel and Nester, 1986), and integrated T-DNAs delivered from such strains often exhibit large deletions (Rossi et al., 1996). VirE2 can coat single-stranded DNA molecules *in vitro* (Gietl et al., 1987; Christie et al., 1988; Citovsky et al., 1988, 1989; Das, 1988; Sen et al., 1989) and has been proposed to coat single-stranded T-DNA molecules (T-strands) and protect them from nucleases as they traffic through the plant cell (Gietl et al., 1987; Citovsky et al., 1988; Tinland et al., 1994; Yusibov et al., 1994). Expression of VirE2 in the plant can complement a *virE2* mutant *Agrobacterium* strain to full virulence (Citovsky et al., 1992; Simone et al., 2001), suggesting that one of VirE2’s functions in transformation occurs in the plant and involves the maintenance of T-DNA integrity (Gietl et al., 1987; Citovsky et al., 1988).

VirE2 has been proposed to assist with nuclear import of T-strands through its interaction with the transcription factor VIP1 (VirE2-interacting protein 1; Tzfira et al., 2001). This observation led to the model that T-DNA-bound VirE2 binds VIP1 and uses VIP1 nuclear localization to deliver T-DNA into the nucleus (the “Trojan Horse” model; Djamei et al., 2007). However, conflicting reports of VirE2 subcellular localization exist in the literature (Citovsky et al., 1992, 1994, 2004; Tzfira and Citovsky, 2001; Tzfira et al., 2001; Li et al., 2005; Bhattacharjee et al., 2008; Grange et al., 2008; Lee et al., 2008; Shi et al., 2014; Lapham et al., 2018). In contrast to the Trojan Horse model, our laboratory showed that VirE2 holds at least a portion of the VIP1 pool outside the nucleus (Shi et al., 2014), and that VIP1 and its homologs are not required for *Agrobacterium*-mediated transformation (Shi et al., 2014; Lapham et al., 2018).

In addition to its proposed structural role in T-strand binding, we investigated other possible functions of VirE2 in transformation. VirE2 interacts with numerous plant proteins (Lee et al., 2008, 2012) including the transcription factors VIP1 and VIP2 (Tzfira et al., 2001; Anand et al., 2007; Pitzschke et al., 2009). We hypothesized that these interactions could lead to changes in plant gene expression, perhaps facilitating transformation. To determine which subcellular site of localization is required for VirE2 to facilitate transformation, we generated plants expressing cytoplasmic localized VirE2-Venus or nuclear localized VirE2-Venus-NLS under the control of a β-estradiol inducible promoter (Zuo et al., 2000). Following induction, these plants were assayed for transformation using a *virE2* mutant *Agrobacterium* strain. Only cytoplasmic localized VirE2 could support transformation, indicating that VirE2’s major function in transformation occurs in the cytoplasm. We also performed RNA-seq and proteomic analyses on transgenic *Arabidopsis thaliana* roots before and after VirE2 expression. Genes previously shown to be important for transformation were differentially expressed in the presence of VirE2, and proteins known to be important for transformation were more prevalent after VirE2 induction, possibly facilitating transformation. Knockout mutant lines of some of the differentially expressed genes (DEGs) exhibited altered transformation phenotypes. Transgenic plants overexpressing cDNAs encoding some of the proteins shown to be more prevalent in the presence of VirE2 had enhanced transformation susceptibility. Taken together, our results suggest that VirE2 alters expression of specific plant genes and proteins to facilitate transformation, and that VirE2’s major role in transformation occurs from its position in the plant cytoplasm.

## Results

### Cytoplasmic but Not Nuclear Localized VirE2 Can Support Transformation

To determine the subcellular localization of VirE2 that is required to facilitate transformation, we first constructed plasmids to express the recombinant proteins VirE2-Venus or VirE2-Venus-NLS [containing a nuclear localization signal (NLS)] constitutively. Tobacco BY-2 protoplasts were individually co-transfected with DNA from each of these constructs and a plasmid containing a red fluorescence protein (RFP) nuclear marker. The protoplasts were imaged 16 h later using confocal microscopy (Supplementary Figure 1). Consistent with data from previous studies (Bhattacharjee et al., 2008; Grange et al., 2008; Lee et al., 2008; Li et al., 2014, 2020; Shi et al., 2014; Li and Pan, 2017; Lapham et al., 2018; Roushan et al., 2018), VirE2-Venus localized to the cytoplasm (Supplementary Figure 1A); however, VirE2-Venus-NLS localized to the nucleus (Supplementary Figure 1B). Although we have not precisely identified where in the cytoplasm VirE2-Venus localizes, it does not localize to the nucleus, and for convenience we shall hereafter refer to the subcellular localization of VirE2-Venus as “cytoplasmic.”

**FIGURE 1:**
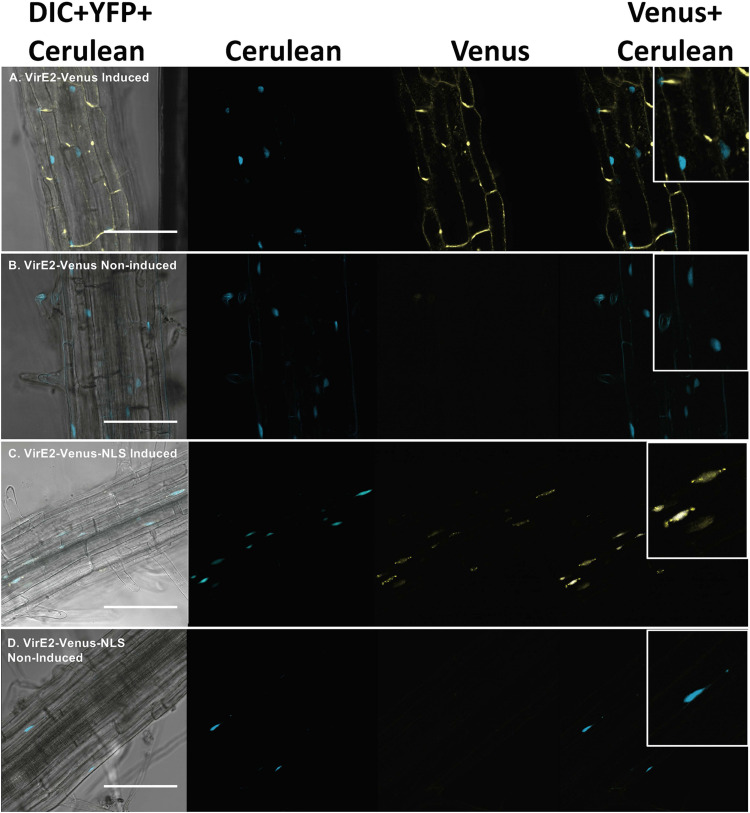
Subcellular localization of VirE2-Venus **(A,B)** and VirE2-Venus-NLS **(C,D)** in *A. thaliana* roots. Transgenic *A. thaliana* plants expressing inducible VirE2-Venus or VirE2-Venus-NLS were treated with β-estradiol **(A,C)** or control solution **(B,D)**. Cerulean-NLS under the control of a CaMV 2 × 35S promoter was used to mark the nuclei. Root cells were imaged by confocal microscopy 9 h after treatment and representative images are shown. Four images of each cell are presented (left to right: Merged DIC + YFP + Cerulean; Cerulean; Venus; merged Venus + Cerulean). Boxes indicate an enlargement of one portion of the merged Venus + Cerulean image. Bars indicate 100 μm.

We generated transgenic *A. thaliana* plant lines expressing either VirE2-Venus or VirE2-Venus-NLS under the control of a β-estradiol inducible promoter (Zuo et al., 2000), and a Cerulean-NLS nuclear marker under the control of a constitutive Cauliflower Mosaic Virus (CaMV) double 35S promoter. After incubating the plants in either control (non-induced) or β-estradiol (induced) solution for 9 h, we imaged the roots using confocal microscopy. Only induced, but not non-induced, roots showed a yellow fluorescence signal (Figures 1A,C), whereas the Cerulean marked nuclei were evident in both non-induced and induced roots (Figures 1A–D). VirE2-Venus localized outside of the nucleus and throughout the cytoplasm (Figure 1A), whereas VirE2-Venus-NLS co-localized with the Cerulean nuclear marker (Figure 1C) in transgenic *Arabidopsis* roots.

**FIGURE 2:**
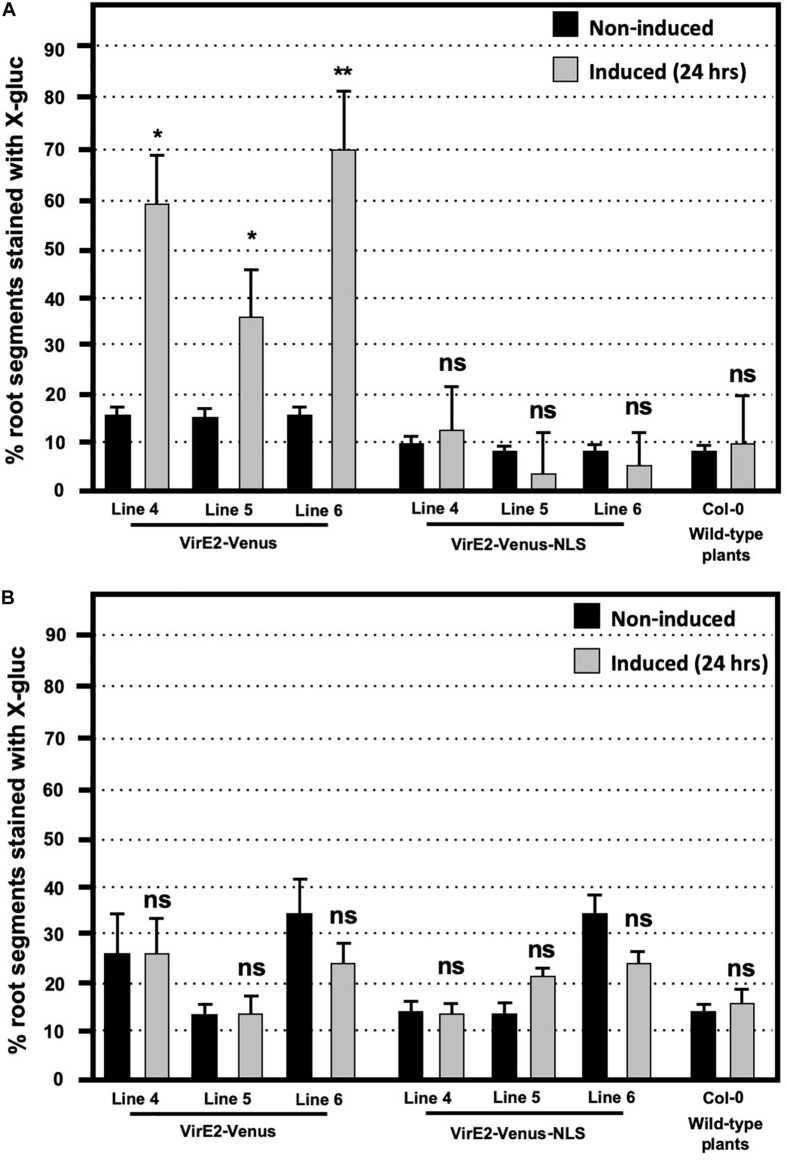
Transformation susceptibility of *Arabidopsis* wild-type (Col-0) and β-estradiol inducible transgenic VirE2-Venus and VirE2-Venus-NLS plants. *Agrobacterium*-mediated transient transformation assays were conducted on roots of three transgenic lines of inducible VirE2-Venus, three transgenic lines of inducible VirE2-Venus-NLS, and wild-type Col-0 plants. Following treatment for 24 h with β-estradiol or control solutions, root segments were inoculated with **(A)** 10^8^ cfu/mL of the *virE2* mutant strain *A. tumefaciens* At1879 containing pBISN2 or **(B)** 10^5^ cfu/mL of the wild-type *VirE2* strain EHA105:pBISN1 (At1529). Root segments were stained with X-gluc 6 days after infection. Bars represent an average of three biological replicates (each replicate containing >60 root segments) + SE. ANOVA test *p-value < 0.05, **p-value < 0.01, ns, not significant.

We performed transient *Agrobacterium*-mediated transformation assays on wild-type (Col-0), and three independent lines each of inducible VirE2-Venus, and inducible VirE2-Venus-NLS transgenic plants. These lines were chosen based upon equivalent levels of expression of the fluorescently tagged VirE2 protein. Plant roots were treated with either control or β-estradiol solution for 24 h before cutting the roots into small segments and infecting them with a *virE2* mutant *Agrobacterium* strain containing the T-DNA binary vector pBISN2 or a *virE2*^+^ control strain containing pBISN1. The T-DNAs of pBISN1 and pBISN2 are identical and contain a plant-active *gusA*-intron gene (Narasimhulu et al., 1996). A low level of transformation was observed in all non-induced samples infected with the *virE2* mutant *Agrobacterium* strain (Figure 2A). Such low-level *virE2*-independent transformation has been observed previously (Stachel and Nester, 1986; Rossi et al., 1996; Dombek and Ream, 1997). Induction of only transgenic plants encoding cytoplasmic-localized VirE2-Venus, but not nuclear-localized VirE2-Venus-NLS, increased transient transformation efficiency compared to that of non-induced levels. The inability of nuclear-localized VirE2-Venus-NLS to complement the *virE2* mutant strain to full virulence was not due to a toxic effect of the protein because both inducible VirE2-Venus and inducible VirE2-Venus-NLS plants showed comparable transformation rates when infected with a *virE2*^+^*Agrobacterium* strain (Figure 2B). In addition, the similar transformation efficiencies of induced VirE2-Venus and VirE2-Venus-NLS plants by a *virE2*^+^*Agrobacterium* strain indicates that nuclear-localized VirE2 does not prevent T-strand uncoating in the nucleus, thus preventing *gusA* transgene expression. Thus, these results indicate that in order for VirE2 to complement the transformation deficiency of a *virE2* mutant *Agrobacterium* strain, it must be localized in the cytoplasm.

### Cytoplasmic-Localized VirE2 Alters Expression of Numerous *Arabidopsis* Genes, Including Those Involved in Defense Response and Transformation Susceptibility

We generated multiple transgenic *A. thaliana* lines expressing untagged *VirE2* under the control of a β-estradiol inducible promoter and tested them for *VirE2* induction by RT-PCR. Root tissue pooled from ∼30 plants of one representative line was harvested after treating either with β-estradiol or control (non-induced) solution for 3 or 12 h. Both the control and β-estradiol solutions contained the avirulent strain *A. tumefaciens* A136 that lacks a Ti-plasmid (Sciaky et al., 1978) at a concentration of 10^8^ cfu/mL. The inclusion of this bacterial strain was done to mimic more closely natural infection conditions because a plant cell will only be exposed to VirE2 in the presence of *Agrobacterium*. RNA was extracted from each sample and induced expression of *VirE2* was confirmed using RT-PCR. VirE2 transcripts were detectable within 1 h of induction (Supplementary Figure 2A). RT-qPCR was performed on samples collected from 3 to 12 h after induction (Supplementary Figure 2B) before submitting the samples for RNA-seq analysis. This analysis was initially performed on one biological replicate composed of roots from ∼30 plants as a pilot study to identify potential target genes to test for transformation phenotypes. DEG were determined using Cufflinks (Trapnell et al., 2012). For this pilot study and considering all time points, a total of 443 *A. thaliana* genes (∼1.5% of the annotated protein coding genome) were differentially expressed in VirE2-induced versus non-induced samples (Supplementary Data Sheet 1).

**FIGURE 3:**
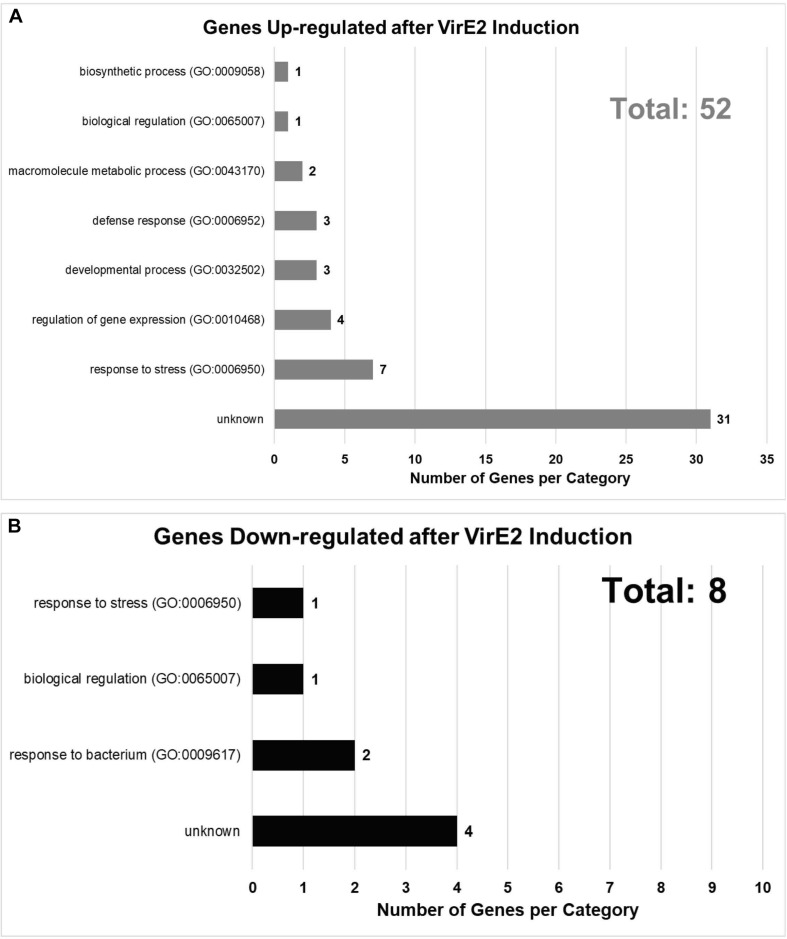
Gene Ontology (GO) Biological Process Categories of up- **(A)** and down-regulated **(B)** genes in the presence of VirE2. Displayed are categories of genes with 1.3-fold or greater change in expression, considering all time points.

We later conducted RNA-seq analysis on two additional biological replicates, each composed of roots from ∼30 plants, of the same inducible *VirE2* line. Using more stringent criteria than used in our pilot study, we identified in total 145 unique up-regulated genes and 25 unique down-regulated genes in induced versus non-induced samples by at least two computational methods with an adjusted *P*-value cut-off of 0.1 across all analyses (Supplementary Data Sheet 2). Of these unique 170 DEGs, 61 were identified in the pilot study (Table 1 and Supplementary Data Sheet 2, yellow highlighted rows). DEGs identified in both studies were displayed according to their annotated Gene Ontology (GO) biological process (Figure 3; Ashburner et al., 2000). Some genes which showed significant changes in expression were tested using RT-qPCR to validate the RNA-seq results. All genes tested by RT-qPCR showed changes in expression consistent with the RNA-seq data (Supplementary Data Sheet 1 and Supplementary Figure 3). Genes involved in response to stress (both biotic and abiotic), regulation of gene expression, biological regulation, and various other developmental, biosynthetic, and metabolic processes were differentially expressed in VirE2-induced plants (Figure 3A). A subset of genes differentially expressed following VirE2 induction are involved in defense responses, particularly those involved in responding to bacteria (Figure 3B).

**TABLE 1:** VirE2 differentially expressed genes in both RNA-seq studies.

Gene ID	Encoded Protein	Up/Down-regulated Second study (Fold-change)	Up/Down-regulated Pilot study (Fold-change)
	VirE2	Up (194.0)-3 h; Up (2342.3)-12 h	Up (188)-3 h; Up (1961.7)-12 h
At1g01580	Ferric reduction oxidase 2	Down (2.5)-3 h; Down (2.4)-12 h	Down (1.4)-3 h; Down (2.5)-12 h
At1g09932	Phosphoglycerate mutase family protein	Up (1.8)	Up (3.6)
At1g14200	RING/U-box superfamily protein SNIPER1	Up (3.4)	Up (1.9)
At1g23730	Beta carbonic anhydrase 3	Up (5.2)	Up (2.8)
At1g26800	E3 ubiquitin-protein ligase MPSR1	Up (3.0)	Up (1.4)
At1g32350	Alternative oxidase 1D	Up (6.9)	Up (2.5)
At1g61560	MILDEW RESISTANCE LOCUS O 6	Up (1.9)	Up (1.7)
At1g61820	Beta-glucosidase 46	Up (1.9)	Up (1.8)
At1g62370	RING/U-box superfamily protein	Up (2.5)	Up (1.9)
At1g63530	Hypothetical protein	Up (3.0)	Up (1.5)
At1g66090	Disease resistance protein (TIR-NBS class)	Up (4.6)	Up (6.3)
At1g73120	F-box/RNI superfamily protein	Down (5.5)	Down (5.7)
At2g16660	Major facilitator superfamily protein	Down (2.4)	Down (3.8)
At2g17040	NAC domain containing protein 36	Up (2.6)	Up (5.0)
At2g23270	Transmembrane protein	Up (2.9)	Up (4.0)
At2g26150	Heat stress transcription factor A-2	Up (11.6)	Up (1.7)
At2g28160	Transcription factor FER-LIKE IRON DEFICIENCY-INDUCED TRANSCRIPTION FACTOR	Up (1.8)	Up (1.4)
At2g29450	Glutathione S-transferase tau 5	Up (2.4)	Up (2.7)
At2g42850	Cytochrome P450, family 718	Up (2.1)	Up (1.4)
At2g44010	Hypothetical protein	Up (1.6)	Up (3.2)
At2g44578	RING/U-box superfamily protein	Up (2.9)	Up (1.6)
At2g45920	U-box domain-containing protein	Up (1.9)	Up (1.4)
At3g07090	PPPDE putative thiol peptidase family protein	Up (2.0)	Up (1.4)
At3g09290	Telomerase activator1 (TAC1)	Up (3.2)	Up (2.2)
At3g09350	Fes1A	Up (3.2)	Up (1.3)
At3g13437	Enhancer of vascular Wilt Resistance 1; EWR1	Up (2.2)	Up (3.6)
At3g14362	DEVIL 19; DVL19; ROTUNDIFOLIA like 10	Up (2.6)	Up (1.7)
At3g15340	Proton pump interactor 2 (PPI2)	Up (2.8)	Up (1.3)
At3g29000	Calcium-binding EF-hand family protein	Up (2.8)	Up (2.6)
At3g48920	Myb domain protein 45	Up (2.1)	Up (3.4)
At3g46810	Cysteine/Histidine-rich C1 domain family protein	Down (2.7)	Down (2.7)
At3g53150	UDP-glucosyl transferase 73D1	Up (2.3)	Up (1.5)
At3g54150	Embryonic abundant protein-like	Up (2.4)	Up (1.8)
At3g61400	1-aminocyclopropane-1-carboxylate oxidase homolog 8	Down (9.7)	Down (2.7)
At4g04990	Serine/arginine repetitive matrix-like protein (DUF761)	Up (2.1)	Up (2.4)
At4g19690	Fe(2+) transport protein 1	Down (2.5)-3 h; Down (2.8)-12 h	Down (2.3)-3 h
At4g26200	1-aminocyclopropane-1-carboxylate synthase 7	Up (4.8)	Up (1.7)
At4g30230	Uncharacterized protein At4g30230	Up (26.2)	Up (2.1)
At4g30960	CBL-interacting serine/threonine-protein kinase 6	Up (1.6)	Up (1.4)
At4g33050	Calmodulin-binding family protein	Up (1.9)	Up (1.3)
At4g34950	Major facilitator superfamily protein	Down (2.3)	Down (3.7)
At4g37290	Transmembrane protein	Up (5.1)	Up (5.7)
At4g39670	ACD11 homolog protein	Up (2.1)	Up (1.5)
At5g02490	Probable mediator of RNA polymerase II transcription subunit 37c	Up (2.3)	Up (2.8)
At5g03545	Expressed in response to phosphate starvation protein	Down (1.6)	Down (2.3)
At5g06760	LEA4-5	Up (8.3)-3 h; Up (4.4)-12 h	Up (6.6)-12 h
At5g13320	Auxin-responsive GH3 family protein	Up (8.3)	Up (27.3)
At5g25450	Cytochrome b-c1 complex subunit 7	Up (2.7)	Up (3.3)
At5g39050	Phenolic glucoside malonyltransferase 1	Up (2.4)	Up (1.4)
At5g39360	EID1-like 2	Up (1.7)	Up (1.5)
At5g39670	Probable calcium-binding protein CML46	Up (2.5)	Up (1.9)
At5g40010	AAA-ATPase ASD, mitochondrial	Up (2.6)	Up (1.3)
At5g43450	1-aminocyclopropane-1-carboxylate oxidase homolog 10	Up (3.9)	Up (3.4)
At5g45840	Phytosulfokin receptor 1	Up (2.5)	Up (1.3)
At5g51440	23.5 kDa heat shock protein, mitochondrial	Up (5.9)	Up (2.1)
At5g54165	Avr9/Cf-9 rapidly elicited protein	Up (4.7)	Up (21.4)
At5g57010	IQ domain-containing protein IQM5	Up (5.4)	Up (1.6)
At5g57510	Cotton fiber protein	Up (4.4)	Up (2.4)
At5g59820	Zinc finger protein ZAT12	Up (2.0)	Up (1.3)
At5g64810	Probable WRKY transcription factor 51	Up (3.0)	Up (1.7)

**FIGURE 4:**
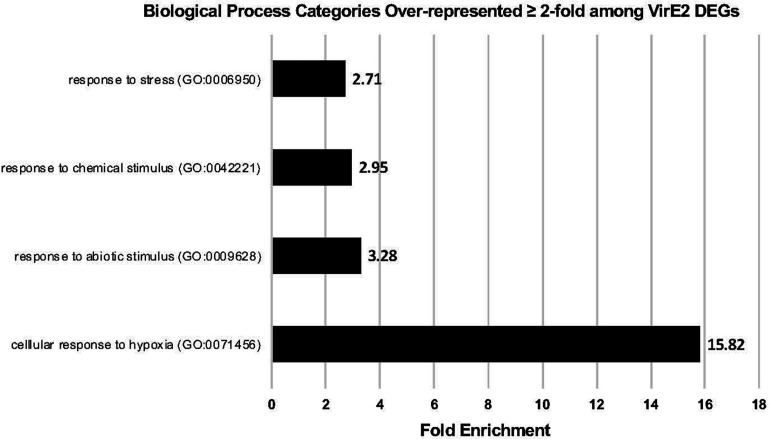
Gene Ontology (GO) Enrichment Analysis of VirE2 differentially expressed genes. GO biological processes of over-represented gene categories for VirE2 differentially expressed genes at all time points. Displayed are results only with a false discovery rate (FDR) < 0.05.

A GO enrichment analysis was performed to determine which categories of genes were over-represented 2-fold or more in the RNA-seq dataset for those 61 DEGs common to both RNA-seq experiments (Figure 4 and Table 1). Genes involved in cellular response to hypoxia, abiotic and chemical stimuli, and stress were enriched. Some stress associated genes, such as those encoding protein phosphatase 2C (down-regulated) and *HEAT SHOCK PROTEIN 90.1* (up-regulated), have previously been shown to be important for transformation (Tao et al., 2004; Park et al., 2014; Supplementary Data Sheets 1, 2). These VirE2-induced changes may facilitate transformation.

It is possible that the differential expression of *Arabidopsis* genes following VirE2 induction is merely a stress response to overexpression of a protein in the plant cytoplasm, as indicated by induction of the *HSP90.1* gene. To control for this possibility, we generated *Arabidopsis* lines that inducibly overexpress *VIP1* (Lapham et al., 2018). *VIP1* encodes a protein that localizes both to the nucleus and the cytoplasm, depending upon the time after osmotic or thigomostimulation (Tsugama et al., 2012, 2014, 2016). Unlike VirE2, VIP1 is not important for *Agrobacterium*-mediated transformation (Shi et al., 2014; Lapham et al., 2018). We conducted RNA-seq analysis of RNA from *Arabidopsis* roots extracted at various times after *VIP1* induction. Supplementary Data Sheet 2 compares VirE2-DEGs with VIP1-DEGs. Under the same conditions (in the presence of *Agrobacterium*, and at the same time point), only two DEG (At3G13437 and At4G26200) overlap between the VirE2 and VIP1 overexpression analyses, and neither of these encode stress-response or heat shock/chaperonin proteins. Thus, overexpression of VirE2 elicits a specific DEG response that differs from that elicited by overexpression of another protein.

### *Arabidopsis* Lines Harboring Mutations in Some Genes Differentially Expressed by VirE2 Exhibit Altered Transformation Phenotypes

Transferred DNA insertion mutant lines of a subset of the VirE2 DEGs identified in our pilot RNA-seq study were tested for transformation susceptibility (Table 2). Transformation results for mutants of VirE2 up-regulated and down-regulated genes are shown in Supplementary Figures 4, 5, respectively, and are summarized in Table 2. If a mutant showed no statistically significant difference in transformation efficiency at any of the tested bacterial concentrations, the results are reported as “No change” in Table 2. However, some of these mutations may still have a minor impact on transformation.

**TABLE 2:** Transformation phenotypes of mutants of VirE2 differentially expressed genes.

Gene Name	Gene_ID	Encoded Protein	Up/Down-regulated (Fold-change)	ABRC Stock ID	Transformation Result
*lncRNA*	At3g12965	Long non-coding RNA	Up (5.8)	SALK_086573	No change
*atpsk3*	At3g44735	Phytosulfokine 3 precursor	Up (5)	SALK_044781	*Decreased transient
*acs6*	At4g11280	1-aminocyclopropane-1-carboxylate synthase 6	Up (3)	SALK_054467	No change
*tst18*	At5g66170	Thiosulfate sulfurtransferase 18	Up (3.7)	CS867285	*Decreased transient and stable
*pr5*	At1g75040	Pathogenesis-related protein 5	Up (14)	SALK_055063C	*Increased transient
*agp14*	At5g56540	Arabinogalactan protein 14	Up (4.9)	SALK_096806	No change
*tasi4*	At3g25795	*Trans*-acting siRNA 4	Up (15.1)	SALK_066997	No change
*miR163*	At1g66725	microRNA 163	Up (3.3)	CS879797	**Decreased stable
*samp*	At2g41380	S-adenosyl-L-methionine-dependent methyltransferases superfamily protein	Up (10.1)	SALK_209995C	No change
*tasi3*	At3g17185	*Trans*-acting siRNA 3	Up (3)	GABI-Kat Stock N432182 (N2051875)	No change
*exl1*	At1g23720	Proline-rich extensin-like family protein 1	Down (3.3)	SALK_010243C	**Decreased stable
*mee39*	At3g46330	Maternal effect embryo arrest 39 (putative LRR receptor-like serine/threonine-protein kinase)	Down (4.7)	SALK_065070C	No change
*rbc3b*	At5g38410	Ribulose bisphosphate carboxylase small chain 3B	Down (7.4)	SALK_117835	No change
*abah3*	At5g45340	Abscisic acid 8′-hydroxylase 3	Down (3.4)	SALK_078170	*Increased transient
*ntr2.6*	At3g45060	High affinity nitrate transporter 2.6	Down (28)	SALK_204101C	*Increased transient
*cup*	At3g60270	Cupredoxin superfamily protein	Down (31.3)	SALK_201444C	**Increased transient
*ntr2:1*	At1g08090	Nitrate transporter 2:1	Down (35.7)	SALK_035429C	*Increased transient
*oep6*	At3g63160	Outer envelope protein 6 (chloroplast)	Down (5.6)	CS862774	*Decreased stable
*esm1*	At3g14210	Epithiospecifier modifier 1	Down (10)	SALK_150833C	**Increased stable
*rld17*	At2g17850	Rhodanese-like domain-containing protein 17	Down (4.7)	SALK_115776C	***Decreased transient and stable
*pp2c25*	At2g30020	Putative protein phosphatase 2C 25	Down (3.5)	SALK_104445	**Increased transient
*adh1*	At1g77120	Alcohol dehydrogenase 1	Down (23.2)	SALK_052699	***Increased transient

ANOVA test *p-value < 0.05, **p-value < 0.01, ***p-value < 0.001.

The *atpsk3*, *tst18*, and *miR163* mutant lines (Table 2 and Supplementary Figures 4B,C,G) showed decreased transformation compared to that of wild-type plants. All three genes are up-regulated in the presence of VirE2 and may therefore facilitate transformation. The *pr5* mutant showed an increase in transient transformation (Table 2 and Supplementary Figure 4D). *PR5* is up-regulated in the presence of VirE2, and because of its role in defense response and effector-triggered immunity (ETI) (Wu et al., 2014) one would predict that the *pr5* mutant would be more susceptible to *Agrobacterium*-mediated infection. At least for transient transformation, this prediction is consistent with our results (Supplementary Figure 4D).

Several of the mutants for genes down-regulated in the presence of VirE2 showed increased transient or stable transformation efficiency compared to that of wild-type plants (Supplementary Figures 5B–F). These genes may act to inhibit transformation, and their VirE2-dependent down-regulation may facilitate transformation, as reflected by the increased susceptibility of their respective knockout mutant lines to *Agrobacterium*-mediated transformation. A *PROTEIN PHOSPHATASE 2C* (Supplementary Figure 5F) was previously identified as a transformation inhibitor (Tao et al., 2004). Conversely, the *exl1*, *oep6*, and *rld17* mutants showed decreased transformation (Table 2 and Supplementary Figures 5A,D,E) even though they are down-regulated in the presence of VirE2. These genes may be important for transformation, but their mechanism of action and regulation during transformation remain unknown.

### The Subcellular Location of VirE2 Results in Different *Arabidopsis* Root Transcriptome Patterns

**FIGURE 5:**
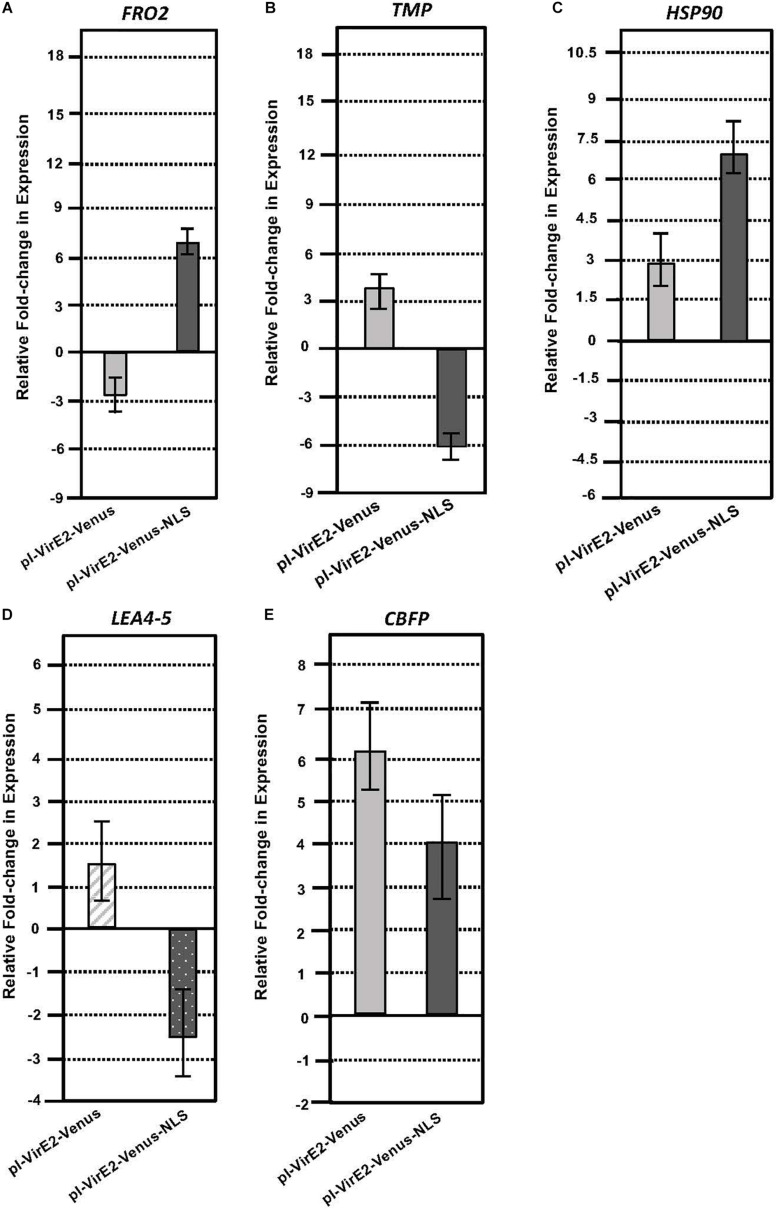
Quantitative RT-PCR analysis of selected VirE2 differentially expressed genes in inducible VirE2-Venus (cytoplasmic) versus inducible VirE2-Venus-NLS (nuclear) plants. VirE2-Venus (left) and VirE2-Venus-NLS (right) results of **(A)** *FRO2*, **(B)** *TMP*, **(C)** *HSP90*, **(D)** *LEA4-5*, and **(E)** *CBFP* gene expression in induced relative to non-induced roots. Bars represent an average of three technical replicates ± SE for one representative biological replicate of one transgenic line. Relative expression is shown after 3 (*LEA4-5* only) or 12 h after induction in the presence of *A. tumefaciens* A136.

Roots of transgenic inducible VirE2-Venus or VirE2-Venus-NLS plants were induced for 3 or 12 h with β-estradiol in the presence of *A. tumefaciens* A136. Total RNA was extracted from infected root samples. A subset of genes which exhibited significant changes in expression after the induction of an untagged VirE2 line (determined by RNA-seq) was tested by RT-qPCR for changes in expression in the inducible VirE2-Venus and inducible VirE2-Venus-NLS samples compared with non-induced samples (Table 3, Figure 5 and Supplementary Datasheets 1, 2). The inducible VirE2-Venus (cytoplasmic localized) samples showed either no change or a similar pattern of gene expression changes to those observed for untagged VirE2 (Table 1 and Figure 5). However, the genes *FERRIC REDUCTION OXIDASE2* (*FRO2*), *TRANSMEMBRANE PROTEIN* (*TMP*), and *LATE EMBRYOGENESIS ABUNDANT 4-5* (*LEA4-5*) showed the opposite pattern of gene expression changes in the VirE2-Venus-NLS (nuclear localized) line compared to that of the VirE2-Venus (cytoplasmic localized) samples (Figures 5A,B,D). These results suggest that the changes in expression of these genes resulted from the cytoplasmic localization of VirE2. Interestingly, both cytoplasmic (VirE2-Venus) and nuclear-localized (VirE2-Venus-NLS) VirE2 caused up-regulation of *HEAT SHOCK PROTEIN 90-1* (*HSP90*) and *CALMODULIN-BINDING FAMILY PROTEIN* (*CBFP*; Figures 5C,E), but to different extents. Up-regulation of these genes occurred after 12 h of induction and could have resulted from downstream effects caused by the presence of VirE2 in the plant regardless of localization. It is also possible that a small amount of VirE2 or VirE2-Venus could have entered the nucleus and was sufficient to induce expression of these two genes.

**TABLE 3:** VirE2 subcellular localization impacts changes in plant gene expression.

Gene Name	Gene_ID	Encoded Protein	Up/Down-Regulated in the presence of VirE2 (untagged)	Up/Down-regulated in the presence of VirE2-Venus (cytoplasmic)	Up/Down-regulated in the presence of VirE2-Venus-NLS (nuclear)
*FRO2*	At1g01580	FERRIC REDUCTION OXIDASE 2	Down 2-fold	Down 2.9-fold	Up 6.3-fold
*TMP*	At4g37290	TRANSMEMBRANE PROTEIN	Up 5-fold	Up 3.9-fold	Down 6.9-fold
*HSP90*	At5g52640	HEAT SHOCK PROTEIN 90-1	Up 6-fold	Up 2.9-fold	Up 7.1-fold
*LEA4-5*	At5g06760	LATE EMBRYOGENESIS ABUNDANT 4-5	Up ≥3-fold	Up 1.6-fold	Down 2.5-fold
*CBFP*	At5g57010	CALMODULIN-BINDING FAMILY PROTEIN	Up 5-fold	Up 6.1-fold	Up 4.0-fold

### VirE2 Alters the *Arabidopsis* Proteome to Facilitate Transformation

**FIGURE 6:**
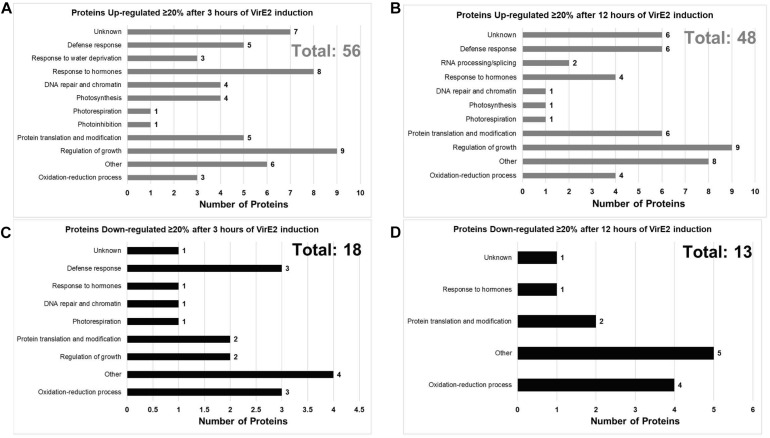
Gene Ontology (GO) Biological Process Categories of VirE2 differentially expressed proteins. Proteins are grouped according to Gene Ontology (GO) process terms. Up-regulated proteins after 3 **(A)** or 12 **(B)** hours of VirE2 induction are shown along with down-regulated proteins after 3 **(C)** or 12 **(D)** hours of VirE2 induction. Only proteins which showed at least a 20% change in abundance for all three biological replicates determined by two different computational methods are shown. Total protein number is shown in the upper right corner of each graph and is highlighted in gray (up-regulated) or in black (down-regulated).

We investigated the effect of VirE2 on the *Arabidopsis* root proteome using the same transgenic inducible *VirE2 Arabidopsis* line that we employed for transcriptome analysis. A total of 135 unique *A. thaliana* proteins (∼0.6% of the detectable proteins) showed a statistically significant change in abundance of at least 20% in all three biological replicates of VirE2-induced samples (Supplementary Datasheet 3). These proteins were graphed according to their annotated GO biological process (Figure 6; Ashburner et al., 2000). Proteins previously shown to be important for transformation, such as histones and histone modifying proteins, arabinogalactan proteins (AGPs), and cyclophilins, showed increased abundance in the presence of VirE2 (Table 4; Supplementary Datasheet 3; Deng et al., 1998; Nam et al., 1999; Gaspar et al., 2004; Crane and Gelvin, 2007; Tenea et al., 2009). These VirE2-induced elevations in protein level likely facilitate transformation. Proteins whose levels changed in the presence of VirE2 did not show changes in their RNA levels (Supplementary Datasheet 3), suggesting that VirE2-induced changes to protein levels occur at the translational or post-translational level.

**TABLE 4:** Proteins previously identified as important for transformation show increased abundance in the presence of VirE2.

Gene ID	Gene Name	Encoded Protein	% Change in Protein Level (Time Post-VirE2 Induction)^*a*^
At2g28740	*HIS4* (formerly *HFO4*)	Histone H4	+37% (3 h); *p* = 0.006
At4g27230	*HTA2*	Histone H2A2	+140% (3 h); *p* = ns
At5g03740	*HD2C* (formerly *HDT3*)	Histone deacetylase 2C	+35% (3 h); *p* = 0.007
At3g44750	*HDA3* (formerly *HDT1*)	Histone deacetylase 3	+50% (12 h); *p* = 0.002
At2g16600	*ROC3*	Rotamase cyclophilin 3	+20% (12 h); *p* = 0.061
At3g56070	*ROC2*	Rotamase cyclophilin 2	+85% (12 h); *p* = 0.046
At1g03870	*FLA9*	FASCICLIN-like arabinogalactan 9	+25% (3 h); *p* = 0.035
At1g28290	*AGP31*	Arabinogalactan protein 31	+50% (3 h); *p* = ns

*^a^p-value according to iBAQ analysis; ns, not significant at p < 0.1.*

We generated transgenic lines of *A. thaliana* that constitutively overexpressed selected genes whose proteins showed increased abundance in response to VirE2-induction (Table 5). Although statistical analysis of the iBAC (intensity-Based Absolute Quantitation) scores showed that the increased ARABINOGALACTAN PROTEIN 31 (AGP31) abundance was statistically significant at only *p* = 0.27, we included this gene in our overexpression analysis because our previous study indicated that AGPs were important for transformation (Gaspar et al., 2004). Roots from multiple T2 generation transgenic lines were assayed for transient and stable transformation susceptibility (Table 5 and Supplementary Figure 6). Some transgenic lines containing a *PEROXIDASE 34* (*PERX34*) overexpressing construct showed decreased stable transformation (Table 5 and Supplementary Figure 6A), whereas most of the *ROTAMASE CYCLOPHILIN 2* (*ROC2*) overexpression lines showed increased transient transformation (Table 5; Supplementary Figure 6B). Transgenic lines containing an overexpression construct for *HISTONE DEACTYLASE 3* (*HDA3*: Supplementary Figure 6C) showed increased transient transformation. Some plant lines containing overexpression constructs for both *HISTONE DEACTYLASE 2C* (*HD2C*: Supplementary Figure 6D) and *AGP31* (Supplementary Figure 6E) also showed increased transformation efficiency. These data suggest that VirE2-induced changes to levels of specific proteins may facilitate transformation.

**TABLE 5:** Transformation phenotypes of *A. thaliana* lines containing overexpression constructs of genes whose proteins show increased abundance post-VirE2 induction.

Gene ID	Gene Name	Encoded Protein	% Change in Protein Level (Time Post-VirE2 Induction)	Transformation Result
At3g49120	*PERX34*	Peroxidase 34	+38% (3 h)	*Decreased stable
At5g03740	*HD2C* (formerly *HDT3*)	Histone deacetylase 2C	+35% (3 h)	**Increased transient and *Increased stable
At3g44750	*HDA3* (formerly *HDT1*)	Histone deacetylase 3	+50% (12 h)	*Increased transient
At3g56070	*ROC2*	Rotamase cyclophilin 2	+85% (12 h)	**Increased transient
At1g28290	*AGP31*	Arabinogalactan protein 31	+50% (3 h)	*Increased transient

ANOVA test *p-value < 0.05, **p-value < 0.01.

Taken together, our results suggest that VirE2 impacts the plant cell on both the RNA and protein levels to facilitate transformation, and that the effect of VirE2 occurs from its position in the plant cytoplasm.

## Discussion

### VirE2 Must Localize to the Plant Cytoplasm to Facilitate Transformation

We have shown that only cytoplasmic localized VirE2-Venus, but not nuclear localized VirE2-Venus-NLS, could complement the loss of virulence of a *virE2*^–^ *Agrobacterium* mutant. These results suggest that the major role of VirE2 in transformation occurs in the cytoplasm.

The reported subcellular localization of VirE2 is controversial. When tagged on its N-terminus, VirE2 was reported to localize to the nucleus (Citovsky et al., 1992, 1994, 2004; Tzfira and Citovsky, 2001; Tzfira et al., 2001; Li et al., 2005). Other studies showed that both N- and C-terminally tagged VirE2 localized to the cytoplasm (Bhattacharjee et al., 2008; Grange et al., 2008; Lee et al., 2008; Shi et al., 2014; Lapham et al., 2018). However, only the C-terminally tagged fusion protein, when expressed in a plant, could complement a *virE2* mutant strain and restore efficient transformation (Bhattacharjee et al., 2008).

More recently, Li et al. (2014) showed that an *Agrobacterium* strain expressing VirE2 with an internal small GFP fragment (GFP11) is virulent. Using this strain and a split-GFP approach, VirE2-GFP11 delivered from *Agrobacterium* could refold with GFP1-10 expressed *in planta* to restore GFP fluorescence. In the plant cell, VirE2-GFP complexes formed filamentous structures mainly in the cytoplasm and with a few that appeared within the nucleus. Roushan et al. (2018) used phiLOV2.1 to tag VirE2 internally and showed that, when transferred from *Agrobacterium*, the protein localized to the cytoplasm of *Arabidopsis* roots and *Nicotiana tabacum* leaves. Li et al. (2020) further demonstrated that only very small amounts of VirE2 could be detected in the nucleus in the presence of VirD2 and T-strands, solving the conundrum of conflicting results from different laboratories. Our results indicate that, regardless of its site of synthesis, only when VirE2-Venus protein localizes to the cytoplasm can it complement a *virE2* mutant *Agrobacterium* strain. An inducible nuclear-localized VirE2-Venus-NLS protein could not complement the *virE2* mutant strain. These results confirm our previous observations (Bhattacharjee et al., 2008) and indicate that VirE2 must localize to the cytoplasm to perform its functions in *Agrobacterium*-mediated transformation.

VirE2 interacts with several *Arabidopsis* importin α (Impα) isoforms in a yeast two hybrid system and in plant cells when overexpressed (Bhattacharjee et al., 2008; Lee et al., 2008). VirE2 interacts with many Impα isoforms in the cytoplasm, but only VirE2-Impa-4 interaction localizes to the nucleus of BY-2 protoplasts. Although VirE2 protein contains two putative bipartite NLS sequences (Citovsky et al., 1992, 1994), structural analyses indicated that the interactions between rice Impα1a and the VirE2 NLS sequences are weak (Chang et al., 2014). Ziemienowicz et al. (2001) observed that VirE2 bound to ssDNA was not imported into isolated tobacco nuclei, but they did observe the import of free VirE2 molecules into the nucleus. On the other hand, VirE2, in addition to the effector protein VirD2, was required for nuclear import of large ssDNA molecules in this *in vitro* system (Ziemienowicz et al., 2001). It is possible that a small amount of VirE2 localizes to the nucleus during transformation. However, based on our results, exclusive nuclear localization of VirE2 does not support transformation.

### VirE2 Alters the *Arabidopsis* Root Transcriptome and Proteome to Facilitate Transformation

We cannot rule out that VirE2 has no function within the nucleus. We therefore investigated possible functions of VirE2 in transformation other than its proposed structural roles in protecting T-strands (Howard and Citovsky, 1990) and/or shaping T-strands to traverse the nuclear pores (Ziemienowicz et al., 2001; Li et al., 2020). VirE2 interacts with the *Arabidopsis* transcription factors VIP1 and VIP2 (Tzfira et al., 2001; Anand et al., 2007; Pitzschke et al., 2009) and various other plant proteins (Lee et al., 2008, 2012). Although VIP1 and its orthologs do not play a role in *Agrobacterium*-mediated transformation (Shi et al., 2014; Lapham et al., 2018), interactions with VIP2 or other proteins could lead to changes in plant gene expression, perhaps facilitating transformation. RNA-seq analysis of transgenic *A. thaliana* roots expressing VirE2 revealed that most transcript abundance changes occurred 12 h post-VirE2 induction (Supplementary Data Sheets 1, 2). Conversely, proteomics analysis indicated that numerous proteins changed abundance 3 h after VirE2 induction, but none of the transcripts for these proteins changed abundance at that early time (Supplementary Data Sheet 3). These results suggest that alterations in mRNA and protein abundance in response to VirE2 expression occur post-transcriptionally, most likely at the translational or post-translational level. This hypothesis is consistent with cytoplasmic- rather than nuclear-localized VirE2. It is also supported by our data showing that proteins involved in translation also exhibited rapid changes in their steady-state levels in response to VirE2 induction (Figure 6). The mechanism by which VirE2 increases the abundance of particular proteins requires further investigation.

Genes involved in plant defense were differentially expressed in response to VirE2 induction (Figure 3 and Supplementary Data Sheets 1, 2). Duan et al. (2018) noted that expression of several defense genes was up-regulated in *A. thaliana* constitutively expressing VirE2 24 h after the plants were treated with the avirulent *Agrobacterium* strain A136. They also found that plants constitutively expressing VirE2 had reduced transformation efficiency compared to wild-type plants. They proposed that this inhibition was caused by enhanced defense responses in the VirE2-expressing plants. We also observed up-regulation of genes involved in innate immune responses 12 h after VirE2 induction in the presence of the avirulent *Agrobacterium* strain A136 (Figure 3 and Supplementary Data Sheet 1, 2), but the genes we identified differed from those identified previously by Duan et al. (2018; Supplementary Data Sheet 1, 2). Ditt et al. (2006) found that genes involved in response to biotic stimulus, abiotic stimulus, and stress were enriched for transcripts up-regulated 48 h after infection of *Arabidopsis* cell cultures (ecotype Ler) by the tumorigenic *Agrobacterium* strain A348. We also observed up-regulation of these same gene categories 12 h after VirE2 induction in the presence of the avirulent *Agrobacterium* strain A136. Veena et al. (2003) observed an increase in defense response gene transcripts early (3–6 h) after *Agrobacterium* infection of *N. tabacum* BY-2 suspension cells, but expression of these genes was suppressed at later infection times (30–36 h) in the presence of *Agrobacterium* strains that could transfer virulence proteins. However, suppression of this delayed defense response did not occur when the plants were infected with the transfer-deficient *Agrobacterium* strain A136 (Veena et al., 2003).

The stress-response associated *ALCOHOL DEHYDROGENASE 1* (*ADH1*) gene was strongly down-regulated in the presence of VirE2 (Table 2; Supplementary Figure 3A and Supplementary Data Sheet 1) and a knockout mutant line of this gene showed increased transformation (Supplementary Figure 5F). Veena et al. (2003) also found that a tobacco alcohol dehydrogenase gene was down-regulated in the presence of a virulent *Agrobacterium* strain at later infection time points. In addition, our RNA-seq experiments revealed that the transcription factor *WRKY33* was up-regulated 12 h after VirE2 induction (Supplementary Data Sheet 2). Zheng et al. (2006) showed that ectopic over-expression of *WRKY33* resulted in increased susceptibility to the bacterial pathogen *Pseudomonas syringae*, and that *WRKY33* could act as a negative regulator of bacterial defense responses.

Genes known to be important for transformation, including those encoding a protein phosphatase 2C (Tao et al., 2004), AGPs (Nam et al., 1999; Gaspar et al., 2004), and heat shock proteins (Park et al., 2014), showed changes in expression in response to VirE2 (Supplementary Data Sheets 1, 2). *PROTEIN PHOSPHATASE 2C 25* (*PP2C25*) was down-regulated by VirE2 (Table 2) and its knockout mutant line exhibited increased transformation (Supplementary Figure 5F). A tomato protein phosphatase 2C (DIG3) was previously shown to act as a negative regulator of transformation by dephosphorylating a serine residue in VirD2 that is critical for VirD2 nuclear import (Tao et al., 2004). VirE2-mediated down-regulation of *PP2C25* may therefore facilitate more efficient nuclear import of VirD2/T-strand complexes.

Induction of VirE2 increased transcript and protein levels of some AGP genes (Supplementary Data Sheets 1, 3). *ARABINOGALACTAN PROTEIN 17* (*AGP17*) was previously shown to be important for transformation by enhancing attachment of *Agrobacterium* to plant cells (Nam et al., 1999; Gaspar et al., 2004). We assayed a knockout mutant of the *AGP14* gene for transformation susceptibility but did not observe any significant difference in transformation compared to wild-type plants (Supplementary Figure 4E). Schultz et al. (2002) identified 50 *Arabidopsis* genes encoding AGPs, and it is plausible that many have redundant functions in the plant cell. *AGP31* showed increased protein levels (although at a *p*-value = 0.27 by iBAQ analysis) in the presence of VirE2 (Table 3 and Supplementary Data Sheet 3) and plants overexpressing *AGP31* exhibited increased transient transformation susceptibility (Table 5 and Supplementary Figure 6E). Therefore, VirE2 may modulate both the transcript and protein levels of some AGPs to facilitate transformation.

Some heat shock protein transcript and protein levels increased in response to VirE2 induction (Supplementary Data Sheet 2), including the transcript encoding *HEAT SHOCK PROTEIN 90* (*HSP90*). Park et al. (2014) demonstrated that over-expression of *HSP90* increased *Arabidopsis* root transformation susceptibility and proposed that HSP90 could act as a molecular chaperone to stabilize VirE2 and other proteins important for transformation. Up-regulation of *HSP90* by VirE2 could also facilitate transformation.

Histones, histone modifying enzymes, and cyclophilins showed increased protein levels in response to VirE2 (Table 4 and Supplementary Data Sheet 3) and have previously been shown, or proposed, to play important roles in transformation (Deng et al., 1998; Nam et al., 1999; Bakó et al., 2003; Crane and Gelvin, 2007; Tenea et al., 2009). Histone H2A2 (HTA2) and histone H4 (HIS4; formerly HFO4) protein levels increased 3 h after induced VirE2 expression (Table 4 and Supplementary Data Sheet 3). Over-expression of *HIS4*, *HTA2*, and some other histone H2A variants increased transformation susceptibility of *Arabidopsis* (Tenea et al., 2009). The histone deacetylases *HD2C* (formerly *HDT3*) and *HDA3* (formerly *HDT1*) also showed increased protein levels in response to VirE2 (Table 4 and Supplementary Data Sheet 3). Crane and Gelvin (2007) showed that RNAi-mediated silencing of *HDA3* and other chromatin-related genes resulted in reduced transformation and T-DNA integration. Plants overexpressing *HDA3* had enhanced transient transformation susceptibility (Table 5 and Supplementary Figure 6C), whereas *HD2C* overexpressing plants had increased transient and stable transformation rates compared to wild-type plants (Table 5 and Supplementary Figure 6D). Increased levels of these histones and histone modifying proteins in response to VirE2 may also facilitate transformation.

VirD2 interacts with various cyclophilin proteins, and this interaction is important for efficient transformation (Deng et al., 1998; Bakó et al., 2003). Two cyclophilin proteins, *ROC2* and *ROC3*, showed increased protein levels post-VirE2 induction (Table 4 and Supplementary Data Sheet 3). Our results also show that plants overexpressing *ROC2* have increased transformation susceptibility (Table 5 and Supplementary Figure 6B). Taken together, these data suggest that VirE2 increases the levels of some cyclophilin proteins, facilitating transformation.

## Conclusion

VirE2 alters the steady-state levels of specific plant RNAs and proteins which are known to be important for transformation. VirE2 likely mediates these changes post-transcriptionally. This model is supported by the rapid changes in levels of certain proteins and more delayed changes in levels of specific RNAs we observed in response to VirE2 induction. Coupled with our observation that cytoplasmic localization of VirE2 is required for it to function in transformation, our results are consistent with a post-transcriptional role in modulating mRNA and protein levels. We conclude that VirE2, from its location in the plant cytoplasm, modulates specific plant steady-state RNA and protein levels post-transcriptionally to facilitate transformation.

## Materials and Methods

### Plasmid and Strain Constructions

Supplementary Table 2 lists the plasmids and strains used in this study. To make a cloning vector with an inducible promoter (Pi), a blunted *Sph*I-*Xho*I fragment containing the *LexA* operator and a minimal CaMV 35S promoter from pER8 (Zuo et al., 2000) was ligated to the blunted *Age*I-*Xho*I plasmid pE3542 to make pE4224.

To make the pPi-VirE2-Venus construction, a *Swa*I-*Not*I fragment containing the VirE2-Venus fragment from pE3759 was cloned into the *Swa*I-*Not*I sites of pE4224 to make Pi-VirE2-Venus (pE4282). The *Asc*I fragment from pE4282 containing the expression cassette pPi-VirE2-Venus and an I-*Sce*I fragment containing P_nos_-Cerulean-NLS from pE4373 were cloned into the *Asc*I and I-*Sce*I sites, respectively, of a binary vector derived from pE4215 containing an XVE expression cassette to make pE4438 (pPZP- Pi-VirE2-Venus-P_nos_-Cerulean-NLS).

To make the pPi-VirE2-Venus-NLS construction, pSAT1-P_35__S_-Venus-VirD2 (pE3561) was digested with *Hin*dIII before self-ligating the backbone fragment to create pSAT1-P_35__S_-Venus-NLS (pE4433). A *Pst*I-*Not*I fragment from pE4433 was used to replace the *Pst*I-*Not*I fragment of pE3759 to make pE4434 (pSAT6-P_35__S_-VirE2-Venus-NLS). A *Swa*I-*Not*I fragment from pE4434 was cloned into the *Sma*I-*Not*I sites of pE4224 to make pE4436 (pSAT1-P_i_-VirE2-Venus-NLS). An *Asc*I fragment containing the Pi-VirE2-Venus-NLS expression cassette from pE4436 was cloned into the *Asc*I site (to replace the Pi-VirE2-Venus expression cassette) of pE4389 to make pE4435. pE4435 was digested with I-*Ceu*I and self-ligated to make pE4439 (pPZP-Pi-VirE2-Venus-NLS-P_nos_-Cerulean-NLS). pE4438 and pE4439 were separately introduced into *A. tumefaciens* GV3101 (Van Larebeke et al., 1974) by electroporation to make *A. tumefaciens* At2155 and At2156, respectively.

To generate a binary vector carrying the Pi-VirE2 expression cassette, a *Swa*I-*Not*I fragment containing the *VirE2* gene from pE4229 was cloned into the *Sma*I-*Not*I sites of pE4224 to create pE4276. The *Asc*I fragment containing pPi-VirE2 was cloned into the *Asc*I sites of pE4215 to generate pE4289. pE4289 was electroporated into *A. tumefaciens* GV3101 to make *A. tumefaciens* At2091.

To generate the constitutive overexpression constructs for proteins whose levels are increased in the presence of VirE2, cDNA clones were ordered from the Arabidopsis Biological Resource Center (ABRC)^1^ for each selected gene (Supplementary Table 2). Each gene was amplified from the cDNA clone using PCR and primers with flanking sequences containing restriction enzyme sites (Supplementary Table 3). Either Phusion High-Fidelity DNA Polymerase (New England Biolabs) or Platinum SuperFi DNA Polymerase (Invitrogen) was used and the reactions were conducted according to the manufacturers’ protocols. The PCR fragments containing *PERX34* (At3g49120) were digested with restriction enzymes which recognized their flanking sequences (Supplementary Table 3) before cloning those fragments into the same sites on pE4297 to create pE4622 (Supplementary Table 2). The blunt-end PCR fragments containing *AGP31* (At1g28290), *HDA3* (At3g44750), *HD2C* (At5g03740), and *ROC2* (At3g56070) were cloned into pBluescript KS+ cut with *Eco*RV to make pE4626, pE4629, pE4633, and pE4637, respectively (Supplementary Tables 2, 3). These plasmids were also sequenced. The *Eco*RI-*Bam*HI fragments from pE4629 (*HDA3*) and pE4637 (*ROC2*) were cloned into the same sites of pE4515 to make pE4630 and pE4638, respectively. The *Sal*I-*Bam*HI fragment from pE4626 (*AGP31*) and the *Bgl*II-*Bam*HI fragment from pE4633 (*HD2C*) were cloned into the same sites of pE4297 to make pE4627 and pE4634, respectively. The *Asc*I fragments containing the overexpression cassettes from pE4622 (*PERX34*), pE4627 (*AGP31*), pE4630 (*HDA3*), pE4634 (*HD2C*), and pE4638 (*ROC2*) were cloned into the *Asc*I site of the binary vector pE4145 to make pE4623, pE4628, pE4631, pE4635, and pE4639, respectively. Each binary vector was electroporated into *A. tumefaciens* GV3101 to make *A. tumefaciens* strains At2259, At2264, At2265, At2267, and At2268, respectively.

### Isolation and Transfection of Tobacco BY-2 Protoplasts

Protoplasts were isolated from tobacco BY-2 cells and transfected as described by Lee et al. (2012). A plasmid encoding a nuclear mRFP marker (pE3170) was co-transfected with the appropriate clones into the protoplasts. Imaging was performed 16 h post-transfection using a Nikon A1R Confocal Laser Microscope System as described in Shi et al. (2014).

### Generation and Selection of Inducible VirE2, VirE2-Venus, VirE2-Venus-NLS, VIP1, and Transgenic *A. thaliana* Plants Constitutively Overexpressing Selected Genes

Wild-type *A. thaliana* plants (ecotype Col-0) were individually transformed by *A. tumefaciens* At2155, At2156, At2091, At2259, At2264, At2265, At2267, or At2268 using a flower dip protocol (Clough and Bent, 1998). T0 generation seeds from the transformed plants were surface sterilized for 15–20 min in a 50% commercial bleach and 0.1% sodium dodecylsulfate (SDS) solution before washing five times with sterile water. After overnight incubation in water at 4°C, the seeds were plated on solidified Gamborg’s B5 medium (Caisson Labs) containing 100 mg/mL Timentin and 20 mg/mL hygromycin. The seeds were placed at 23°C under a 16/8-h light/dark cycle. T1 generation hygromycin-resistant seedlings for the inducible lines were transplanted to soil and grown under the same temperature and light conditions. For inducible VirE2 plants, hygromycin was used to select for homozygous plants. Homozygous T2 plants containing the inducible VirE2-Venus and VirE2-Venus-NLS constructions were used for future experiments. T1 generation hygromycin-resistant seedlings for each of the constitutive overexpression lines were transferred to baby food jars containing solidified B5 medium for 10–14 days. Roots of each plant were cut into 3–5 mm segments and assayed as described in Tenea et al. (2009). Root segments were infected with *A. tumefaciens* At849 [GV3101:pMP90 (Koncz and Schell, 1986) containing pBISN1 (Narasimhulu et al., 1996)] to measure transient transformation at a concentration of 10^6^cfu/mL. Shoots were re-rooted in solidified B5 medium in the jars for 7–10 days before transferring plantlets to soil.

Transgenic plants overexpressing VIP1 were generated using *A. tumefaciens* At2082 as previously described (Lapham et al., 2018).

### Imaging of VirE2-Venus and VirE2-Venus-NLS Transgenic *A. thaliana* Roots

Inducible VirE2-Venus and VirE2-Venus-NLS seedlings (T2 generation) were germinated on B5 medium containing 100 mg/mL Timentin and 20 mg/mL hygromycin. The seedlings were transferred after 2 weeks to plates containing B5 medium lacking antibiotics. These plates were placed vertically in racks to promote root growth on the surface of the medium. After 10 days, the plates were placed horizontally and B5 liquid medium containing 10 μM β-estradiol dissolved in DMSO (β-estradiol solution) or B5 plus DMSO only (control solution) was pipetted onto the surface until a thin layer covered the root tissue (4–5 mL). The roots were incubated in the solution for 9 h before imaging using a Nikon A1R Confocal Laser Microscope System as described in Shi et al. (2014).

### Assaying Inducible VirE2-Venus and VirE2-Venus-NLS Transgenic *A. thaliana* Roots for Complementation of *virE2*^–^ Mutant *Agrobacterium*

Three transgenic lines of Inducible VirE2-Venus (Lines #4-6) and VirE2-Venus-NLS (Lines #4-6) seedlings (T2 generation) were grown and treated with either 10 μM β-estradiol induction or control solution for 24 h as described above. Root segments were infected as described in Tenea et al. (2009) using either *A. tumefaciens* At1529 or the *virE2*^–^ mutant strain At1879 at a concentration of 10^6^ or 10^8^ cfu/mL, respectively (Supplementary Table 2). Three replicates were assayed for each line with root segments pooled from 10 to 30 plants for each replicate. A total of 80 or more root segments were scored for each data point and statistical analysis was performed using ANOVA.

### VirE2, VirE2-Venus, VirE2-Venus-NLS, and VIP1 Induction in the Presence of *Agrobacterium*

Inducible VirE2 (line #10) or inducible VIP1 (line #12) T3 generation plants were grown and assayed as described above, except that *A. tumefaciens* A136 (lacking a Ti plasmid) were added either to induction (1 μM β-estradiol) or control solution at a concentration of 10^8^ cfu/mL. Roots from 30 plants were cut after 0, 3, or 12 h treatment, rinsed with sterile water, dried on a paper towel, and frozen in liquid nitrogen before RNA extraction.

Inducible VirE2-Venus Line #4 and inducible VirE2-Venus-NLS Line #4 T2 generation plants were also grown, treated, and harvested in the same manner as the inducible VirE2 plants before isolating RNA for quantitative RT-PCR (RT-qPCR) analysis.

### Preparation of Samples for RNA-seq Analysis and Quantitative RT-PCR

For both RNA-seq and RT-qPCR analyses, RNA was isolated from non-induced and induced roots in the presence of *Agrobacterium* after 0, 3, and 12 h of treatment using TriZol reagent^2^. Three biological replicates of inducible VirE2 *A. thaliana* transgenic line #10 were analyzed by both RNA-seq and RT-qPCR. The inducible VIP1 *A. thaliana* transgenic line #12 was analyzed by RNA-seq and two biological replicates were analyzed by RT-qPCR (Lapham et al., 2018). Two biological replicates of inducible VirE2 Venus transgenic line #4 and inducible VirE2-Venus-NLS transgenic line #4 were analyzed by RT-qPCR. cDNA was made from polyA^+^ RNA using an Illumina TruSeq Stranded mRNA kit without rRNA depletion. One biological replicate was sequenced at the Purdue Genomics Core Facility on an Illumina HiSeq 2500 DNA sequencer using single-end, 100 cycle rapid run chemistry for the initial VirE2 pilot study and the VIP1 study. RNA from two additional VirE2 biological replicates was similarly sequenced by the Cornell University Institute of Biotechnology Genomics Facility, using an Illumina TruSeq-3′ RNA-seq kit to make cDNA.

A total of 2 μg of total RNA was treated with Ambion DNase I (Thermo Fisher Scientific) before submitting the RNA for sequencing. For RT-qPCR, cDNA was synthesized from 1.45 μg of total RNA treated with Ambion DNase I using SuperScript III reverse transcriptase (Thermo Fisher Scientific) following the manufacturer’s protocols. RT-qPCR was performed using FastStart Essential Green Master reagents (Roche) on a Roche LightCycler 96. Primer sequences for gene amplification are listed in Supplementary Table 3. RT-qPCR data were analyzed using the LightCycler 96 software and Microsoft Excel.

### RNA-seq Bioinformatic Analysis: Pilot Study

RNA was submitted to the Purdue Genomics Core Facility for sequencing after treatment with DNase I to remove any contaminating genomic DNA. Ribosomal RNA was depleted and cDNA libraries (stranded) were prepared from each of the samples before sequencing. Between 15 and 23 million reads were obtained for each sample (100 nucleotides per read) which were quality trimmed and mapped to the *A. thaliana* genome using TopHat (Trapnell et al., 2010). DEGs were determined from the mapped (bam) files using Cuffdiff from the Cufflinks suite of programs (Trapnell et al., 2010). Custom perl scripts were used to extract genes for which fold-changes of 3 or greater occurred between the induced and non-induced control samples at their respective time points. The resulting genes were annotated by hand and separated into categories based on their GO functions which were found in the National Center for Biotechnology Information (NCBI) database^3^.

### RNA-seq Bioinformatic Analysis by Purdue Bioinformatics Core: Second Study

Sequence quality was assessed using FastQC (v 0.11.7)^4^ for all samples and quality and adapter trimming was done using TrimGalore (0.4.4) (Krueger, 2017) to remove the sequencing adapter sequences and bases with Phred33 scores less than 30. The resulting reads of length >25 bases were retained (original read length = 50 and lib type = unstranded) respectively. The quality trimmed reads were mapped against the reference genome using STAR (Dobin et al., 2013) (v 2.5.4b). STAR derived mapping results and annotation (GTF/GFF) file for reference genome were used as input for HTSeq (Anders et al., 2015) package (v 0.7.0) to obtain the read counts for each gene feature for each replicate. Counts from all replicates were merged using custom Perl scripts to generate a read count matrix for all samples.

The merged counts matrix was used for downstream differential gene expression analysis. Genes that did not have counts in all samples were removed from the count matrix and genes that had counts in some samples but not in others were changed from 0 to 1 in order to avoid having infinite values calculated for the fold change. Differential gene expression (DEG) analysis between treatment and control was carried out using ‘‘R’’ (v 3.5.1)^5^ with two different methods (DESeq2 and edgeR). Basic exploration of the read count data file such as accessing data range, library sizes, etc. was performed to ensure data quality. An edgeR object was created by combining the count’s matrix, library sizes, and experimental design using the edgeR (Robinson et al., 2010) (v 3.24.3) package. Normalization factors were calculated for the count’s matrix, followed by estimation of common dispersion of counts. An exact test for differences between the negative binomial distribution of counts for the two experimental conditions resulted in finding differential expression, which was then adjusted for multiple hypothesis testing. DESeq2 (Love et al., 2014) (v 1.22.2) was also used to find DEGs. Both use an estimate variance-mean test based on a model using the negative binomial distribution. The significant genes were identified by examining the adjusted *p*-value.

Additionally, STAR mapping (bam) files were used for analysis by the Cuffdiff from Cufflinks (v 2.2.1) (Trapnell et al., 2010) suite of programs which perform DE analysis based on FPKM values. Cuffdiff uses bam files to calculate Fragments per Kilobase of exon per Million fragments mapped (FPKM) values, from which differential gene expression between the pairwise comparisons can be ascertained. DEG lists detected by at least two or more methods (DESeq2, edgeR, and Cufflinks) were generated using custom Perl scripts.

Gene annotations were retrieved from BioMart databases using biomartr package in “R.” The “transcript_biotype,” “description” attributes were extracted using mart = “plants_mart” and dataset = “athaliana_eg_gene.” GO enrichment analysis was also performed using DEGs from two or more methods while using two replicates. Singular Enrichment Analysis (SEA) from agriGO (Du et al., 2010) was used to perform GO enrichment analysis (count = 5 with Fisher exact *t*-test with multiple testing). A GO enrichment analysis was performed using the PANTHER Classification system and online tools^6^.

### Genotyping and *Agrobacterium*-Mediated Transient and Stable Transformation Assays of T-DNA Insertion Lines

*Arabidopsis thaliana* T-DNA insertion lines tested in this study are listed in Table 2. Seeds for these lines were obtained from the ABRC (see text footnote 1). For genotyping, DNA was isolated from leaves sampled from 10 to 15 individual plants after freezing the tissue in liquid nitrogen and grinding it into a fine powder using a sterile tube pestle. A total of 0.5 mL of extraction buffer (100 mM Tris pH 8.0, 50 mM EDTA, 500 mM NaCl) was added to the ground tissue before mixing thoroughly. A total of 26 μL of 20% SDS solution was added to each sample before mixing by inverting the tubes. The samples were incubated in a 65°C water bath for 20 min and were mixed by inverting every 5 min during the incubation. After removing the samples from the water bath, 125 μL of potassium acetate buffer was added to each sample before mixing. The potassium acetate buffer is made by mixing 60 mL of 5 M KOAc from crystals, 11.5 mL glacial acetic acid, and 28.5 mL of filtered H_2_O to make 100 mL (3 M of potassium and 5 M of acetate in the final solution). The tubes were placed on ice for up to 20 min before centrifugation at top speed for 10 min in a microcentrifuge at 4°C. The supernatant solution was transferred to a fresh tube (∼600 μL). The samples were centrifuged a second time if cellular debris were still evident within the supernatant solution. A 0.7 volume (420 μL) of isopropanol was added to the supernatant fluid before mixing the samples and placing them at −20°C for at least 1 h to precipitate the DNA. The samples were centrifuged at top speed for 10 min in a microcentrifuge at 4°C to pellet the DNA. The DNA pellets were washed with 500 μL of 70% ethanol by flicking the tube until the pellets released from the bottom of the tube. The samples were centrifuged again for 5 min before carefully removing the ethanol. The pellets were then allowed to air-dry for 5–10 min before resuspending the pellets in 30 μL of 1 xTE buffer (10 mM Tris–Cl, 1 mM EDTA [pH 8.0]) plus 20 μg/mL RNase A.

Lines homozygous for the annotated T-DNA insertions were confirmed by PCR (primer sequences are listed in Supplementary Table 3). PCR reaction mixes were made using ExTaq Buffer (TaKaRa), dNTPs (0.2 mM), the appropriate forward and reverse primers (0.2 μM each), homemade Taq polymerase, and water with a tenth volume of sample added to act as a template. The reactions were incubated at 95°C for 3 min before performing 35 cycles of a 30 s, 95°C denaturation step, followed by a 30 s annealing step (temperature was ∼5°C lower than the average melting temperature for each primer set), and a 1 min, 72°C extension step (1 min). A final 10 min extension step at 72°C followed the last cycle before PCR products were visualized using gel electrophoresis.

*Arabidopsis thaliana* plants homozygous for their annotated T-DNA insertion were grown for 20 days in baby food jars containing sterile Gamborg’s B5 medium before cutting their roots into 3–5 mm segments. The segments were assayed as described in Tenea et al. (2009). *A. tumefaciens* At849 (GV3101:pMP90 containing pBISN1) was used to measure transient transformation, whereas *A. tumefaciens* A208 (Sciaky et al., 1978) was used for stable transformation (Supplementary Table 2). Three replicates were assayed for each experiment with root segments from 10 plants pooled for each replicate. A minimum of 80 root segments were scored for each data point and statistical analysis was performed using ANOVA.

### Protein Isolation and Proteomics Analysis

Roots were homogenized in 8 M urea using a Percellys^®^ 24 homogenizer (Bertin) and incubated at room temperature for 1 h with continuous vortexing before centrifugation at 14,000 rpm for 15 min at 4°C. The supernatant solution was transferred to a new tube and the protein concentration was determined using a Pierce^TM^ BCA assay (Thermo Fisher Scientific). A total of 100 μg protein from each sample (equivalent volume) was taken for digestion. Proteins were first precipitated using four volumes of cold acetone (−20°C) overnight before centrifugation at 14,000 rpm for 15 min at 4°C to collect the precipitated proteins. Protein pellets were washed twice with 80% cold (−20°C) acetone, dried in a speed-vac for 5 min, and then solubilized in 8 M urea. Samples were reduced using 10 mM dithiotreitol and cysteine alkylated using 20 mM iodoacetamide. This was followed by digestion using sequence grade Lyc-C/Trypsin (Promega) mix at a 1:25 (enzyme:substrate) ratio to enzymatically digest the proteins. All digestions were carried out at 37°C overnight. The samples were cleaned over C18 MicroSpin columns (Nest Group), dried, and resuspended in 97% purified H_2_O/3% acetonitrile (ACN)/0.1% formic acid (FA). After BCA at the peptide level, 1 μg of each sample was loaded onto the column.

Digested samples were analyzed using a Dionex UltiMate 3000 RSLC Nano System coupled with a Q Exactive^TM^ HF Hybrid Quadrupole-Orbitrap Mass Spectrometer (Thermo Scientific, Waltham, MA, United States). Peptides were first loaded onto a 300 μm × 5 mm C18 PepMap^TM^ 100 trap column and washed with 98% purified water/2% acetonitrile (ACN)/0.01% formic acid (FA) using a flow rate of 5 μL/min. After 5 min, the trap column was switched in-line with a 75 μm × 50 cm reverse phase Acclaim^TM^ PepMap^TM^ RSLC C18 analytical column heated to 50°C. Peptides were separated over the analytical column using a 120 min method at a flow rate of 300 nL min^–1^. Mobile phase A contained 0.01% FA in purified water while mobile phase B consisted of 0.01% FA/80% ACN in purified water. The linear gradient began at 2% B and reached 10% B in 5 min, 30% B in 80 min, 45% B in 91 min, and 100% B in 93 min. The column was held at 100% B for the next 5 min before returning to 5% B where it was equilibrated for 20 min. Samples were injected into the QE HF through the Nanospray Flex^TM^ Ion Source fitted with an emitter tip from New Objective. MS spectra were collected from 400 to 1600 m/z at 120,000 resolution, a maximum injection time of 100 ms, and a dynamic exclusion of 15 s. The top 20 precursors were fragmented using higher-energy C-trap dissociation (HCD) at a normalized collision energy of 27%. MS/MS spectra were acquired in the Orbitrap at a resolution of 15,000 with a maximum injection time of 20 ms. The raw data were analyzed using MaxQuant software (v. 1.5.3.28) against a TAIR 10 protein database combined with VirE2 proteins (Cox and Mann, 2008; Cox et al., 2011, 2014). The search was performed with the precursor mass tolerance set to 10 ppm and MS/MS fragment ions tolerance was set to 20 ppm. The enzyme was set to trypsin and LysC, allowing up to two missed cleavages. Oxidation of methionine was defined as a variable modification, and carbamidomethylation of cysteine was defined as a fixed modification. The “unique plus razor peptides” (razor peptides are the non-unique peptides assigned to the protein group with the most other peptides) were used for peptide quantitation. The false discovery rate (FDR) of peptides and proteins identification was set at 0.01. iBAQ scores and MS/MS counts for each identified protein were compared between the non-induced and induced samples. Proteins which showed a 0.2-fold (20%) increase or decrease in abundance in the induced versus non-induced samples for at least two biological replicates by comparing both iBAQ scores and MS/MS counts were considered to have levels which changed in response to VirE2 induction.

## Data Availability Statement

The RNA-seq data presented in this study can be found in online repositories. The names of the repository/repositories and accession number(s) can be found below: NCBI GEO with the accession number GSE172314.

## Author Contributions

SG and RL designed the research and wrote the manuscript with input from all authors. RL, L-YL, and SG analyzed data. All authors performed the experiments.

## Conflict of Interest

The authors declare that the research was conducted in the absence of any commercial or financial relationships that could be construed as a potential conflict of interest.

## Footnotes

1 www.arabidopsis.org

2 http://www.thermofischer.com

3 https://www.ncbi.nlm.nih.gov/

4 https://www.bioinformatics.babraham.ac.uk/projects/fastqc/

5 http://www.r-project.org/

6 http://geneontology.org/docs/go-enrichment-analysis/
