# Supplementary material for: Agrobacterium VirE2 Protein Modulates Plant Gene Expression and Mediates Transformation From Its Location Outside the Nucleus
Source: Front Plant Sci. 2021 Jun 4;12:684192. doi: 10.3389/fpls.2021.684192 (PMC8213393; doi:10.3389/fpls.2021.684192)
Supplement: Supplementary file 1 [file Data_Sheet_1.zip › Supplemental Figures.PDF]

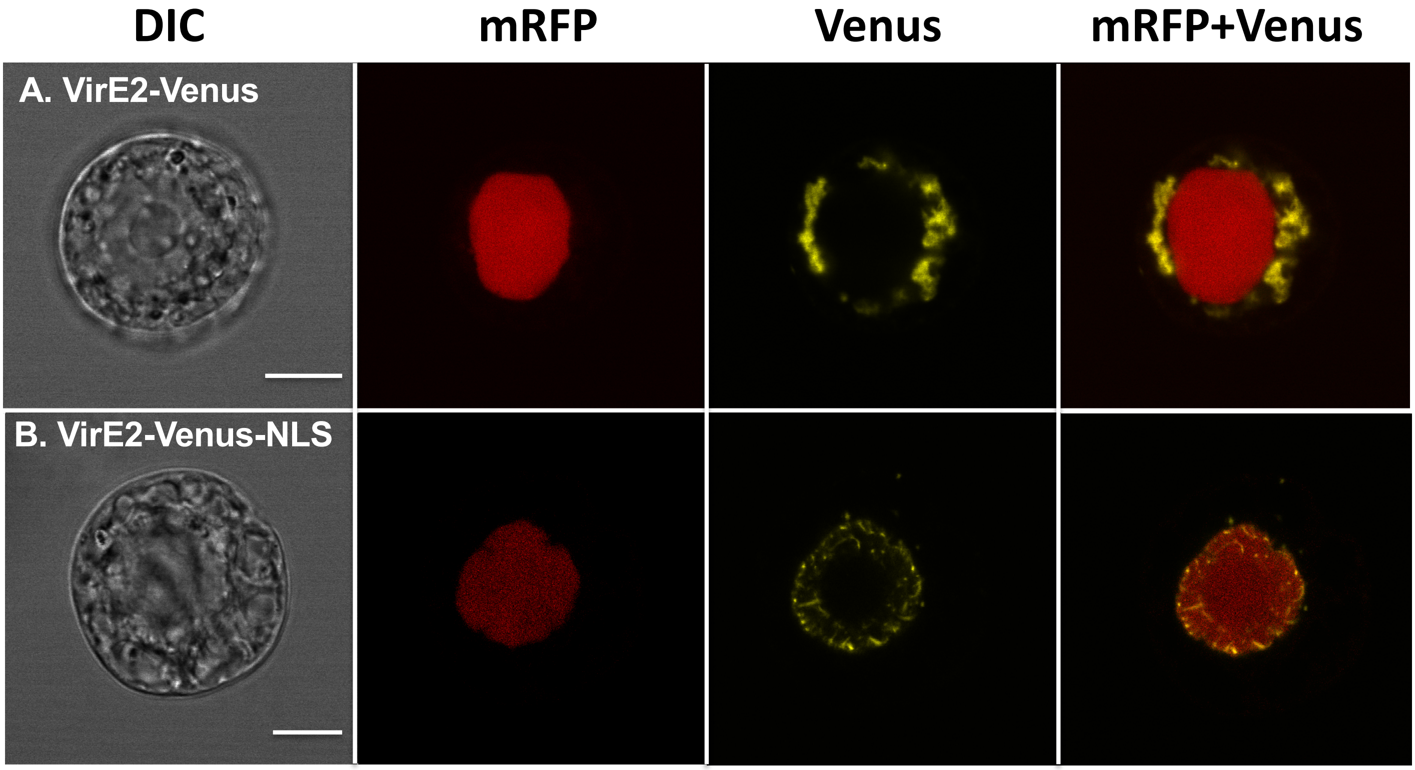

Supplemental Figure 1. Subcellular localization of VirE2-Venus (A) and VirE2-Venus-NLS (B) in tobacco BY-2 protoplasts. A total of 10 µg of DNA encoding VirE2-Venus or VirE2-Venus-NLS was co-transfected with 10 µg of DNA encoding a nuclear marker mRFP-NLS into tobacco BY-2 protoplasts. Cells were imaged by confocal microscopy 16 hr after transfection and representative images are shown. Four images of each cell are presented (left to right: DIC; mRFP; YFP; merged YFP + mRFP). We examined at least ten cells per experiment and performed each experiment three times. We saw the same localization patterns each time. Bars indicate 10 µm.

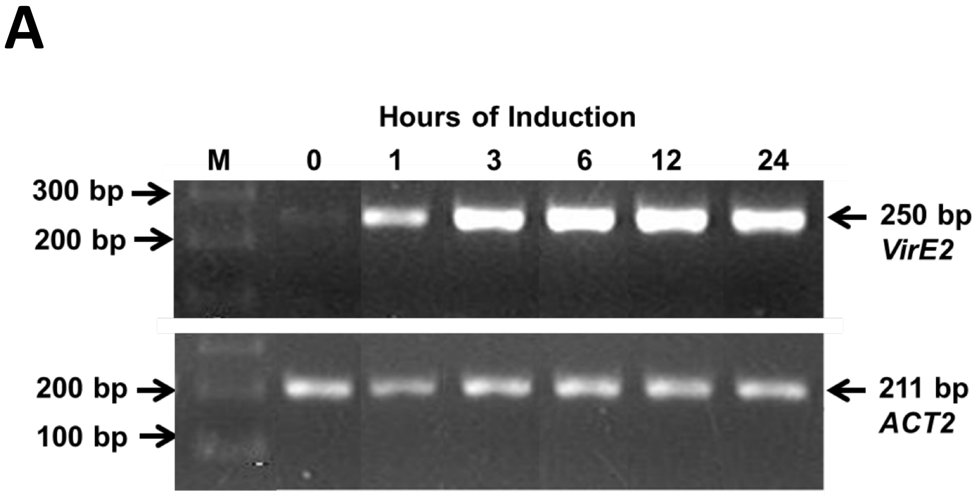

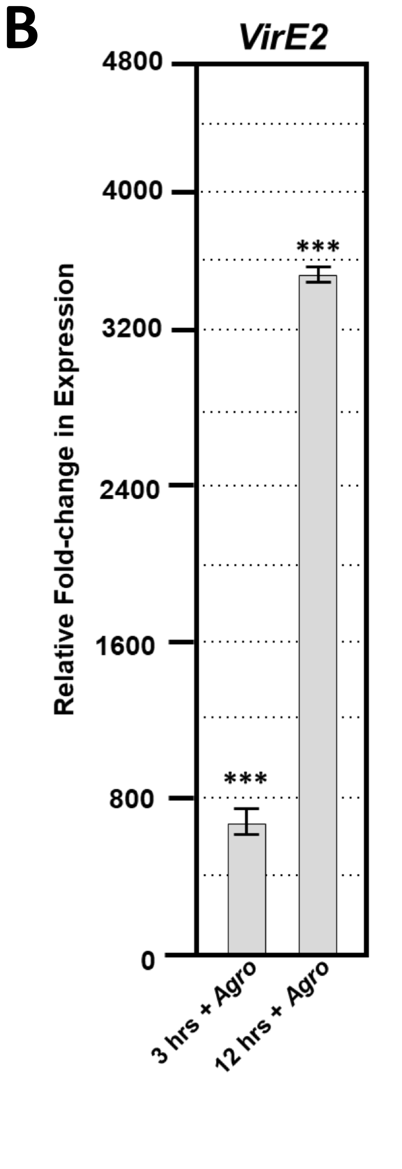

Supplemental Figure 2. Expression kinetics of *VirE2* measured by RT and RT-qPCR . (**A**) A 250 bp PCR product was amplified from the 3' end of *VirE2* transcripts and visualized by ethidium bromide staining after electrophoresis through a 1.5% agarose gel. Samples were harvested 0, 1, 3, 6, 12, and 24 h post-induction with β-estradiol. As a control for RNA integrity, a 211 bp PCR product was amplified from *ACTIN2* (*ACT2*) transcripts. M, size marker; (**B**) Quantitative RT-PCR of *VirE2* gene expression in induced relative to non-induced roots in the presence of *A. tumefaciens* A136. Results show the average of three technical replicates ± SE. Relative expression is shown after 3 and 12 hr. ANOVA test: *P-value < 0.05, **P-value < 0.01, ***P-value <0.001.

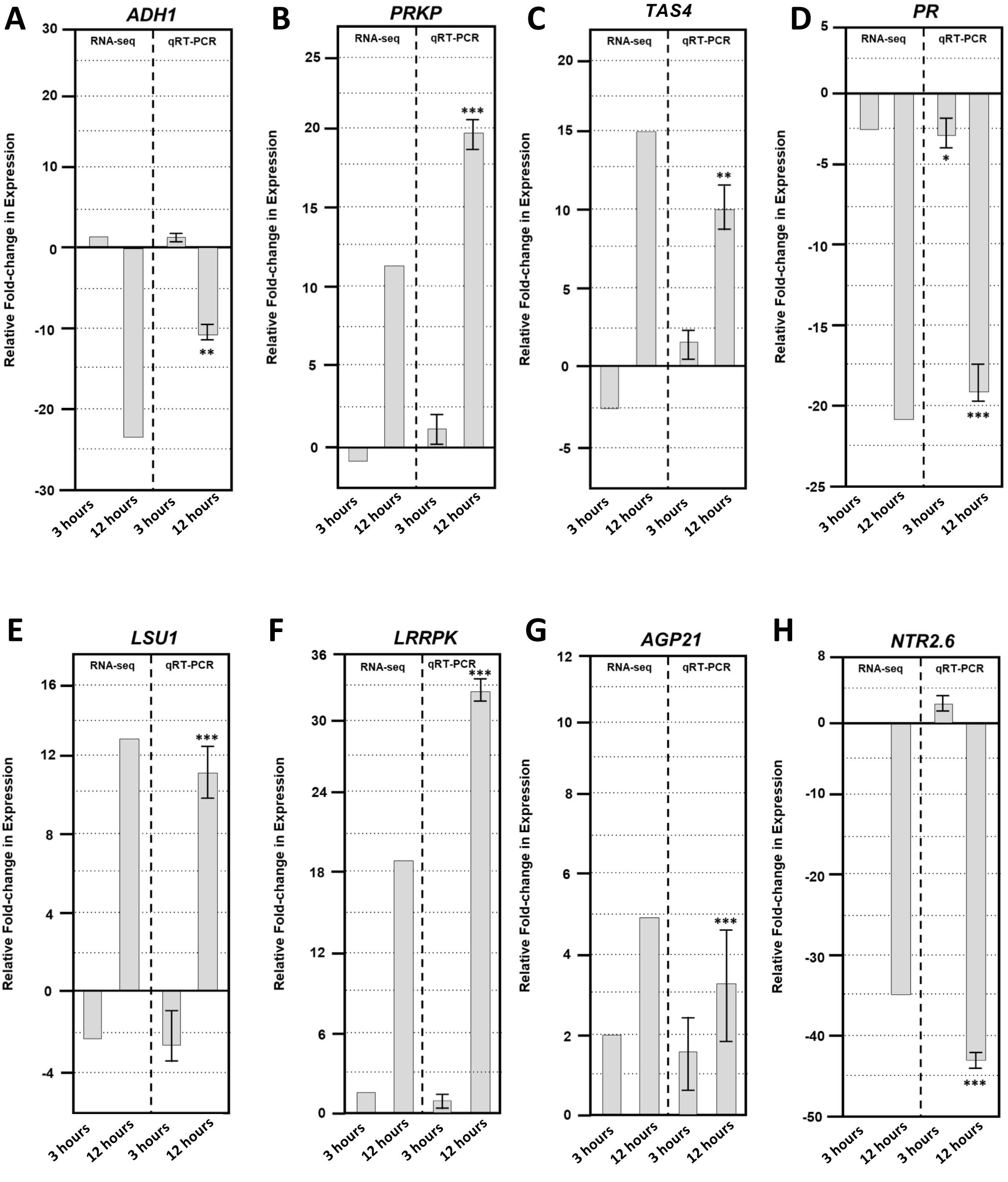

Supplemental Figure 3. Quantitative RT-PCR of selected VirE2 Differentially Expressed Genes. RNA-seq (left) and quantitative RT-PCR (right) results of (**A**) *ADH1* (**B**) *PRKP* (**C**) *TAS4*, (**D**) *PR,* (**E**) *LSU1*, (**F**) *LRRPK,* (**G**) *AGP21*, and (**H**) *NTR2.6* gene expression in induced relative to non-induced roots. Results represent an average of three replicates ± SE for inducible *VirE2* Line #10. Relative expression is shown 3 and 12 hours after induction in the presence of *A. tumefaciens* A136. ANOVA test: *P-value < 0.05, **P-value < 0.01, ***P-value <0.001.

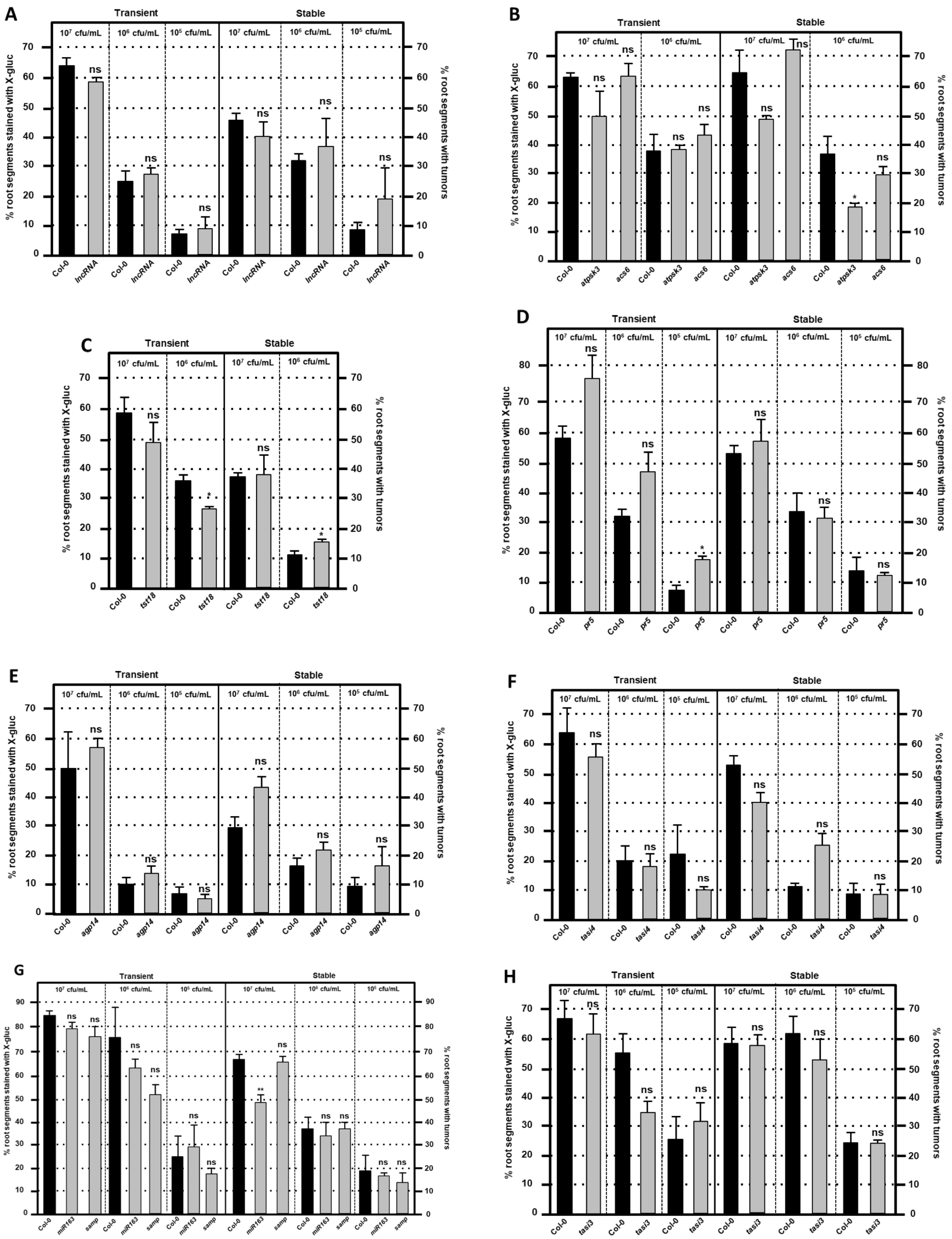
A
Transient
Stable
10⁷ cfu/mL
10⁶ cfu/mL
10⁵ cfu/mL
% root segments stained with X-gluc
% root segments with tumors
Col-0
lncRNA
ns
B
Transient
Stable
10⁷ cfu/mL
10⁶ cfu/mL
% root segments stained with X-gluc
% root segments with tumors
Col-0
atpsk3
acs6
ns
*
C
Transient
Stable
10⁷ cfu/mL
10⁶ cfu/mL
% root segments stained with X-gluc
% root segments with tumors
Col-0
tst18
ns
*
D
Transient
Stable
10⁷ cfu/mL
10⁶ cfu/mL
10⁵ cfu/mL
% root segments stained with X-gluc
% root segments with tumors
Col-0
pr5
ns
*
E
Transient
Stable
10⁷ cfu/mL
10⁶ cfu/mL
10⁵ cfu/mL
% root segments stained with X-gluc
% root segments with tumors
Col-0
agp14
ns
F
Transient
Stable
10⁷ cfu/mL
10⁶ cfu/mL
10⁵ cfu/mL
% root segments stained with X-gluc
% root segments with tumors
Col-0
tasi4
ns
G
Transient
Stable
10⁷ cfu/mL
10⁶ cfu/mL
10⁵ cfu/mL
% root segments stained with X-gluc
% root segments with tumors
Col-0
miR163
samp
ns
**
H
Transient
Stable
10⁷ cfu/mL
10⁶ cfu/mL
10⁵ cfu/mL
% root segments stained with X-gluc
% root segments with tumors
Col-0
tasi3
ns

Supplemental Figure 4. Transformation susceptibility of *Arabidopsis* wild-type (Col-0) and T-DNA insertion mutant plants of VirE2 up-regulated genes

Agrobacterium-mediated transient (left) or stable (right) transformation assays were conducted on Col-0, *lncRNA* (A), *atpsk3*, *acs6* (B), *tst18* (C), *pr5* (D), *agp14* (E), *tasi4* (F), *miR163*, *samp* (G), and *tasi3* (H) mutant plants. Root segments were inoculated with 10^7, 10^6, or 10^5 cfu/mL of *A. tumefaciens* At849 (transient) or A208 (stable). For the transient assay, the root segments were stained with X-gluc 6 days after infection. For stable transformation, tumors were scored 30 days after infection. Numbers represent an average of three biological replicates (each replicate containing >60 root segments) $\pm$ SE. ANOVA test *Pvalue < 0.05, **Pvalue < 0.01, ns: not significant. The data are shown only if the transformation efficiency was ≥ 5%.

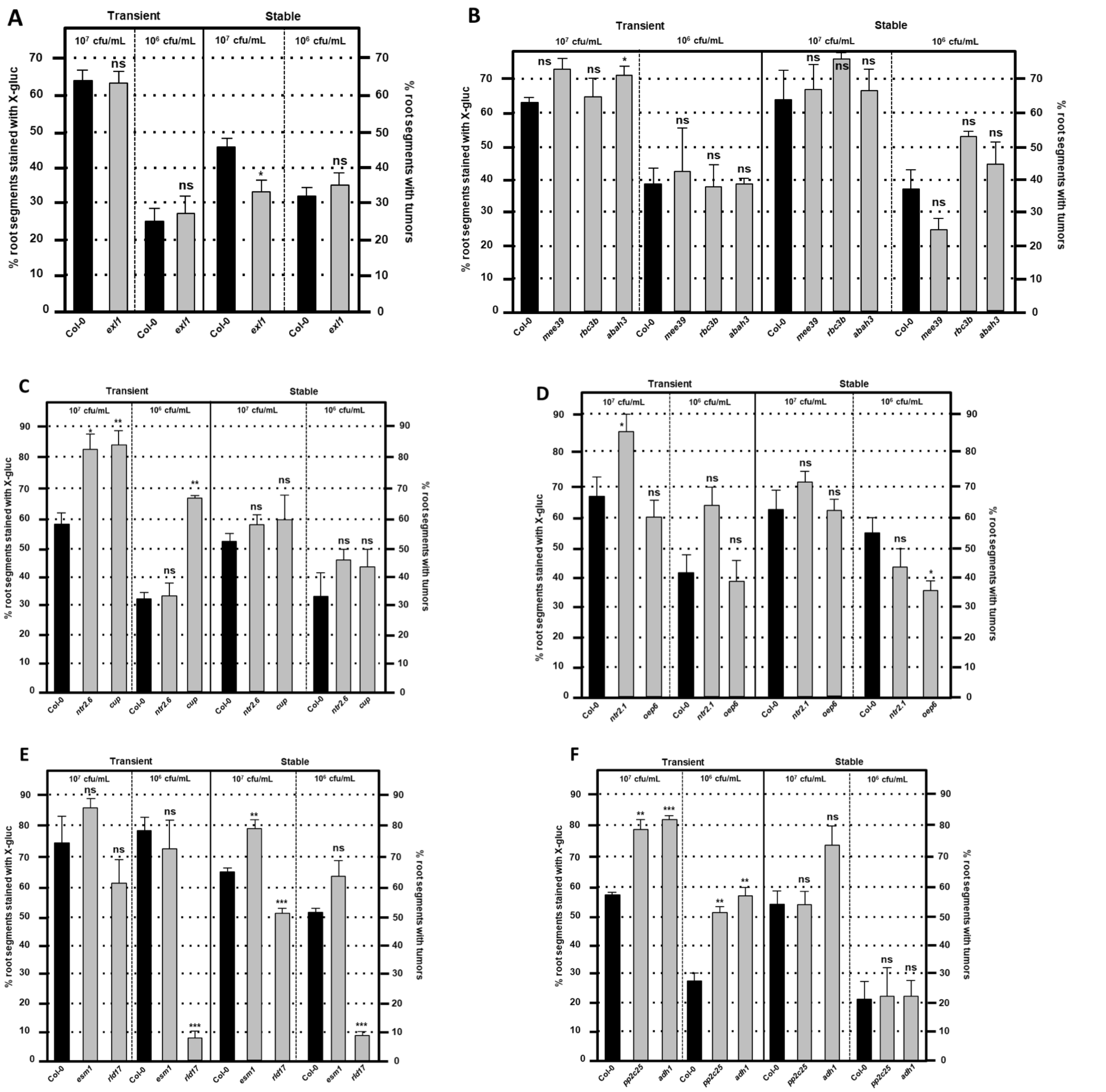

Supplemental Figure 5. Transformation susceptibility of *Arabidopsis* wild-type (Col-0) and T-DNA insertion mutant plants of VirE2 down-regulated genes.

Agrobacterium-mediated transient (left) or stable (right) transformation assays were conducted on Col-0, *exl1* (A), *mee39*, *rbc3b*, *abah3* (B), *ntr2.6*, *cup* (C), *ntr2.1*, *oep6* (D), *esm1*, *rld17* (E), *pp2c25*, and *adh1* (F) mutant plants. Root segments were inoculated with 10^7 or 10^6 cfu/mL of *A. tumefaciens* At849 (transient) or A208 (stable). For the transient assay, the root segments were stained with X-gluc 6 days after infection. For stable transformation, tumors were scored 30 days after infection. Numbers represent an average of two or three biological replicates (each replicate containing > 60 root segments) ± SE. ANOVA test *Pvalue < 0.05, **Pvalue < 0.01, ***Pvalue < 0.001, ns: not significant.

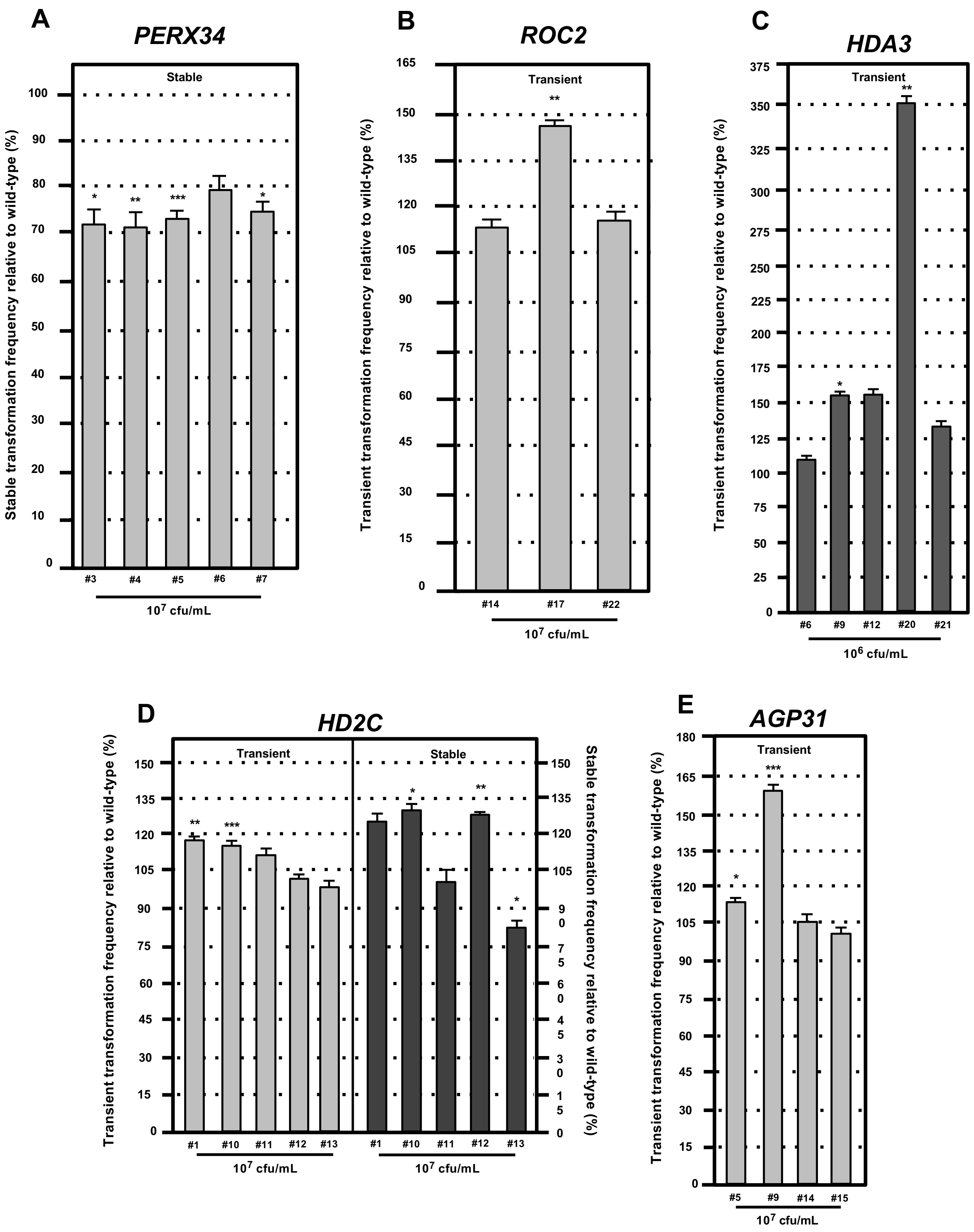
A
PERX34
Stable
Stable transformation frequency relative to wild-type (%)
#3
#4
#5
#6
#7
10^7 cfu/mL
B
ROC2
Transient
Transient transformation frequency relative to wild-type (%)
#14
#17
#22
10^7 cfu/mL
C
HDA3
Transient
Transient transformation frequency relative to wild-type (%)
#6
#9
#12
#20
#21
10^6 cfu/mL
D
HD2C
Transient
Stable
Transient transformation frequency relative to wild-type (%)
Stable transformation frequency relative to wild-type (%)
#1
#10
#11
#12
#13
10^7 cfu/mL
E
AGP31
Transient
Transient transformation frequency relative to wild-type (%)
#5
#9
#14
#15
10^7 cfu/mL

Supplemental Figure 6. Transformation susceptibility of *Arabidopsis* overexpression plants of genes whose protein levels are increased in response to VirE2 relative to wild-type (Col-0).

Agrobacterium-mediated transformation assays were conducted on Col-0, *PERX34* (**A**), *ROC2* (**B**), *HDA3* (**C**), *HD2C* (**D**), and *AGP31*

(**E**) overexpression plants. Numbers of the x-axis represent independent transgenic over-expression lines (T2 generation). Root segments were inoculated with 10^7 cfu/ml or 10^6 cfu/mL of *A. tumefaciens* At849 (transient) and A208 (stable). For the transient assay, the root segments were stained with X-gluc 6 days after infection. For stable transformation, tumors were scored 30 days after infection. Bars represent an average of three biological replicates (each replicate containing >60 root segments) $\pm$ SE. ANOVA test *Pvalue < 0.05, **Pvalue < 0.01, ***Pvalue < 0.001.
