# Supplementary material for: Agrobacterium VirE2 Protein Modulates Plant Gene Expression and Mediates Transformation From Its Location Outside the Nucleus
Source: Front Plant Sci. 2021 Jun 4;12:684192. doi: 10.3389/fpls.2021.684192 (PMC8213393; doi:10.3389/fpls.2021.684192)
Supplement: Supplementary file 1 [file Data_Sheet_1.zip › Supplemental Tables.pdf]

Supplemental Table 1. VirE2 differentially expressed genes tested using RT-qPCR.

Gene Name	Gene_ID	Encoded Protein
ADH1	At1g77120	Alcohol dehydrogenase 1
PRKP	At1g51840	Protein kinase-related protein
lncRNA	At3g25795	Trans-acting siRNA 4
PR	At4g33720	Putative pathogenesis-related protein
LSU1	At3g49580	Response to low sulfur 1
LRRPK	At1g51830	Putative leucine-rich repeat protein kinase
AGP21	At1g55330	Arabinogalactan protein 21
NTR2.6	At3g45060	High affinity nitrate transporter 2.6

Supplemental Table 2. Bacterial strains used in this study.

Strain name	Description	Antibiotic resistance[a]	Reference or source
	***E. coli* strains**		
DH10B	F$^-$ mcrA Δ(*mrr-hsd*RMS-*mcr*BC) Φ80d*lac*ZΔM15 Δ*lac*X74 *end*A1 *rec*A1 *deo*R Δ(*ara,leu*)7697 *ara*D139 *gal*U *gal*K *nup*G *rps*L λ$^-$	None	Durfee et al., 2008
TOP10	F- *mcrA* Δ(*mrr-hsd*RMS-*mcr*BC) Φ80*lac*ZΔM15 Δ *lac*X74 *rec*A1 *ara*D139 Δ(*araleu*)7697 *gal*U *gal*K *rps*L (StrR) *end*A1 *nup*G	None	Invitrogen
Stable	F' *proA+B+ lacI^q Δ(lacZ)M15 zzf::Tn10* (TetR) Δ(*ara-leu*) *7697 araD139 fhuA* Δ*lacX74 galK16 galE15 e14- Φ80dlacZΔM15 recA1 relA1 endA1 nupG rpsL* (StrR) *rph spoT1* Δ(*mrr-hsdRMS-mcrBC)*	None	New England Biolabs
E886	pBluescript (pBS) II KS (+) in DH5α	Amp	Stratagene
E3542	pSAT1-Venus-C	Amp	Lee et al., 2008
E3561	pSAT1-P_{35S}-Venus-VirD2	Amp	Lee et al., 2008
E3759	pSAT6-VirE2-Venus	Amp	Lee et al., 2008
E4145	pPZP-RCS2-P_{ocs}-*hpt*II-RI	Spec	Lee et al., 2012; This study
E4215	T-DNA binary vector XVE- *hptII*	Spec	Lapham et al., 2018
E4223	T-DNA binary vector XVE-P_{nos}-mCherry-*ABD2-hptII*	Spec	This study
E4224	pSAT1-Inducible Promoter (minimal 35S-LexA operator; pI)	Amp	Lapham et al., 2018; This study
E4229	pSAT5-P_{35S}-VirE2	Amp	Lee et al., 2008
E4276	pSAT1-pI-VirE2	Amp	This study
E4282	pSAT1-pI-VirE2-Venus	Amp	This study
E4288	T-DNA binary vector XVE-inducible VIP1	Spec	Lapham et al., 2018; This study
E4289	T-DNA binary vector XVE-inducible VirE2	Spec	This study
E4292	T-DNA binary vector XVE-inducible VirE2-Venus- P_{nos}-mCherry-*ABD2-hptII*	Spec	This study
E4297	pSAT1A-P_{35S}-Multi-cloning Site (MCS)-T_{35S}	Amp	Lee et al., 2008
E4372	pSAT5-P_{35S}-mCherry-ABD2	Amp	This study
E4373	pSAT4-P_{nos}-Cerulean-VirD2NLS	Amp	Lee et al., 2008
E4375	pSAT4-P_{nos}-Cerulean-SV40NLS	Amp	Lee et al., 2008
E4376	T-DNA binary vector XVE-inducible VirE2-Venus- P_{nos}-mCherry-*ABD2-hptII*-P_{nos}-Cerulean-SV40NLS	Spec	This study
E4377	T-DNA binary vector XVE-inducible VirE2-Venus-*hptII*-P_{nos}-Cerulean-SV40NLS	Spec	This study

E4380	T-DNA binary vector XVE-inducible VirE2-Venus- P_{35S}-mCherry-*ABD2-hptII*-P_{nos}-Cerulean-SV40NLS	Spec	This study
E4386	T-DNA binary vector XVE-inducible VirE2-Venus- P_{35S}-mCherry-*ABD2-hptII*	Spec	This study
E4389	T-DNA binary vector XVE-inducible VirE2-Venus- P_{35S}-mCherry-*ABD2-hptII*- P_{nos}-Cerulean-VirD2NLS	Spec	This study
E4433	pSAT1-P_{35S}-Venus-VirD2NLS	Amp	This study
E4434	pSAT6-P_{35S}-VirE2-Venus-VirD2NLS	Amp	This study
E4435	T-DNA binary vector XVE-inducible VirE2-Venus-VirD2NLS- P_{35S}-mCherry-*ABD2-hptII*-P_{nos}-Cerulean-VirD2NLS	Spec	This study
E4436	pSAT1-pI-VirE2-Venus-VirD2NLS	Amp	This study
E4438	T-DNA binary vector XVE-inducible VirE2-Venus-*hptII*- P_{nos}-Cerulean-VirD2NLS	Spec	This study
E4439	T-DNA binary vector XVE-inducible VirE2-Venus-VirD2NLS-*hptII*- P_{nos}-Cerulean-VirD2NLS	Spec	This study
E4515	pSAT1-P_{35S}-MCS-T_{35S}	Kan	Lee et al., 2008; This study
E4594	*PERX34*: DKLAT3G49120 cDNA clone	Spec	ABRC*
E4597	*AGP31*: DKLAT1G28290 cDNA clone	Spec	ABRC*
E4601	*HDA3*: DKLAT3G44750 cDNA clone	Spec	ABRC*
E4602	*HD2C*: DKLAT5G03740 cDNA clone	Spec	ABRC*
E4603	*ROC2*: DKLAT3G56070 cDNA clone	Spec	ABRC*
E4622	pSAT1A-P_{35S}-*PERX34*-T_{35S}	Amp	This study
E4623	pPZP-P_{35S}-*PERX34*-T_{35S}- P_{ocs}-*hptII*-RI	Spec	This study
E4626	pBS-*AGP31*	Amp	This study
E4627	pSAT1A- P_{35S}-*AGP31*-T_{35S}	Amp	This study
E4628	pPZP-P_{35S}-*AGP31*-T_{35S}- P_{ocs}-*hptII*-RI	Spec	This study
E4629	pBS-*HDA3*	Amp	This study
E4630	pSAT1- P_{35S}-*HDA3*-T_{35S}	Kan	This study
E4631	pPZP-P_{35S}-*HDA3*-T_{35S}- P_{ocs}-*hptII*-RI	Spec	This study
E4633	pBS-*HD2C*	Amp	This study
E4634	pSAT1A- P_{35S}-*HD2C*-T_{35S}	Amp	This study
E4635	pPZP-P_{35S}-*HD2C*-T_{35S}- P_{ocs}-*hptII*-RI	Spec	This study
E4637	pBS-*ROC2*	Amp	This study
E4638	pSAT1- P_{35S}-*ROC2*-T_{35S}	Kan	This study
E4639	pPZP-P_{35S}-*ROC2*-T_{35S}- P_{ocs}-*hptII*-RI	Spec	This study

Strain name	Description	Antibiotic resistance[a]	Reference
	***Agrobacterium* strains**		
A208	Tumorigenic; pTiT37 in A136	Rif	Sciaky et al., 1978
EHA105	Non-tumorigenic, disarmed pTiBO542 without Kan gene in A136	Rif	Hood et al., 1993
GV3101	Non-tumorigenic, disarmed pTiC58 in C58 background	Rif, Gent	Koncz and Schell, 1986
At2	Non-tumorigenic; A136	Rif	Sciaky et al., 1978
At849	pBISN1 in GV3101	Rif, Gent, Kan	Narasimhulu et al., 1996
At1529	pBISN1 in EHA105	Rif, Kan	This study
At1879	pBISN2 in EHA105 with in-frame deletion of *virE2*	Rif, Kan, Spec	This study
At2082	pE4288 in GV3101	Rif, Gent, Spec	Lapham et al., 2018
At2091	pE4289 in GV3101	Rif, Gent, Spec	This study
At2155	pE4438 in GV3101	Rif, Gent, Spec	This study
At2156	pE4439 in GV3101	Rif, Gent, Spec	This study
At2259	pE4623 in GV3101	Rif, Gent, Spec	This study
At2264	pE4628 in GV3101	Rif, Gent, Spec	This study
At2265	pE4631 in GV3101	Rif, Gent, Spec	This study
At2267	pE4635 in GV3101	Rif, Gent, Spec	This study
At2268	pE4639 in GV3101	Rif, Gent, Spec	This study

[a]Amp, ampicillin; Gent, gentamicin; Kan, kanamycin; Rif, rifampicin; Spec, spectinomycin

*ABRC: Arabidopsis Biological Resource Center, The Ohio State University (www.arabidopsis.org)

References for Supplemental Table 2

Citovsky, V., Lee, L.-Y., Vyas, S., Glick, E., Chen, M.-H., Vainstein, A., Gafni, Y., Gelvin, S.B. and Tzfira, T. (2006). Subcellular localization of interacting proteins by bimolecular fluorescence complementation *in planta*. *J. Mol. Biol*. 362, 1120– 1131.

Durfee, T., Nelson, R., Baldwin, S., Plunkett, G., Burland, V., Mau, B., Petrosino, J.F., Qin, X., Muzny, D.M., Ayele, M., Gibbs, R.A., Csörgo, B., Pósfai, G., Weinstock, G.M., and Blattner, F.R. (2008). The complete genome sequence of *Escherichia coli* DH10B: Insights into the biology of the laboratory workhorse. *J. Bacteriol*. 190, 2597-2606.

Hood, E.E., Gelvin, S.B., Melchers, L.S., and Hoekema, A. (1993). New *Agrobacterium* helper plasmids for gene transfer to plants. *Transgen. Res*. 2, 208-218.

Koncz, C., and Schell, J. (1986). The promoter of T_L-DNA gene *5* controls the tissue-specific expression of chimaeric genes carried by a novel type of *Agrobacterium* binary vector. *Mol. Gen. Genet*. 204, 383-396.

Lapham, R., Lee L.-Y., Tsugama D., Lee S., Mengiste T., and Gelvin S.B. (2018). *VIP1* and its homologs are not required for *Agrobacterium*-mediated transformation, but play a role in *Botrytis* and salt stress responses. *Frontiers Plant Sci*. 9, 1-15.

Lee, L.-Y., Fang, M.-J., Kuang, L.-Y., and Gelvin, S. B. (2008). Vectors for multi-color bimolecular fluorescence complementation to investigate protein-protein interactions in living plant cells. *Plant Methods* 4, 24.

Lee, L.-Y., Wu, F.-H., Hsu, C.-T., Shen, S.-C., Yeh, H.-Y., Liao, D.-C., Fang, M.-J., Liu, N.-T., Yen, Y.-C., Dokládal, L., Sýkorová, E., Gelvin, S.B., and Lin, C.-S. (2012). Screening of a cDNA library for protein-protein interactions directly in planta. *Plant Cell* 24, 1746-1759.

Narasimhulu, S.B., Deng, X.-B., Sarria, R., and Gelvin, S.B. (1996). Early transcription of *Agrobacterium* T-DNA genes in tobacco and maize. *Plant Cell* 8, 873-886.

Sciaky, D., Montoya, A. L., and Chilton, M.-D. (1978). Fingerprints of *Agrobacterium* Ti plasmids. *Plasmid* 1, 238-253.

Supplemental Table 3. Primer sequences used in this study.

Primer Name	Sequence (5' to 3')	Tm (°C)	Purpose
VirE2 qPCR Fwd	CTTGGTGAAGCAGCTGACAAATACTC	58	RT-qPCR
Universal qPCR Rev	AGACTGGTGATTTTTGCGGACTCTAG	58.6	RT-qPCR
ADH1 (At1G77120) qPCR Fwd	CGGGGTTGTGGAAAAGTACATGAAC	58.2	RT-qPCR
ADH1 (At1G77120) qPCR Rev	GCTTCAAGCACCCATGGTGATG	59	RT-qPCR
PRKP (At1G51840) qPCR Fwd	TGACCCGAACTTCGACCTTTACC	58.8	RT-qPCR
PRKP (At1G51840) qPCR Rev	TCAATGAACCGCTTTGAGTAGCGTATAC	58.6	RT-qPCR
TAS4 (At3G25795) qPCR Fwd	AAGTCACTCAAACACTGACGTGAACC	59.1	RT-qPCR
TAS4 (At3G25795) qPCR Rev	CGTCCTTCACCACGGCAATTTCATG	60.6	RT-qPCR
PR (AtT4G33720) qPCR Fwd	CACTATACTCAGGTTGTGTGGAGAAACTC	58.3	RT-qPCR
PR (At4G33720) qPCR Rev	CCACTCGCCAACCCAGTTAC	58.3	RT-qPCR
LSU1 (At3G49580) qPCR Fwd	GAGCTGGAGGTCGAGTCTTTAGAAC	58.5	RT-qPCR
LSU1 (At3G49580) qPCR Rev	CTTATTCTACGAGGAAGAGACGACAGAAG	57.7	RT-qPCR
LRRPK (At1G51830) qPCR Fwd	TCCTTCATCAGCTAGAAGACCGAACATG	59.7	RT-qPCR
LRRPK (At1G51830) qPCR Rev	CCGAGCCAATGGGGTCACTTC	60.6	RT-qPCR
AGP21 (At1G55330) Geno Fwd	AAAGATCTATGGAGGCAATGAAGATG	55	RT-qPCR

AGP21 (At1G55330) Geno Rev	TTCTTAAGTCAAAAGATGAAACCAGATGC	56	RT-qPCR
AtNTR2.6 (At3G45060) qPCR Fwd	GAAGAGCATTACTATGGAGCGGAATGG	59	RT-qPCR
AtNTR2.6 (At3G45060) qPCR Rev	CTTCACTAGACATGAGCCGGAGATC	58.4	RT-qPCR
FRO2 (At1G01580) qFwd	CTGCATTTTGGAGAAAGACCTAATCTCAAG	57.7	RT-qPCR
FRO2 (At1G01580) qRev	AGAGTTATATACGCAATCACCAGCTGAAAC	58.5	RT-qPCR
TMP (At4G37290) qFwd	GAGTCGTCCGCTTGGTCTAAC	57.5	RT-qPCR
TMP (At4G37290) qRev	CTTGGACCTGAGTGCTTAACAAATCG	58	RT-qPCR
HSP90 (At5G52640) qFwd	GCTAGGATTCACAGGATGTTGAAGTTG	57.6	RT-qPCR
HSP90 (At5G52640) qRev	ACTTCCTCCATCTTGCTCTCTTCAG	58.1	RT-qPCR
LEA4-5 (At5G06760) qFwd	GTCGGACAACCGCTCATAACAC	58.2	RT-qPCR
LEA4-5 (At5G06760) qRev	AGAACAAGTGAACAACACCGTTTATCC	57.6	RT-qPCR
CBFP (AT5G57010) qFwd	ACAAGTCAACCTTTCTCCTCGTGTAG	58.3	RT-qPCR
CBFP (At5G57010) qRev	GCTTGGAAGACCCATGCAAGATAG	57.9	RT-qPCR
Left Border Primer (SALK)	TGGTTCACGTAGTGGGCCATCG	61.5	T-DNA insertion line genotyping
12965 lncRNA Geno Fwd (SALK_086573)	AAGAGCTCCTAGCTATATATTCTGGAGACTC	58	T-DNA insertion line genotyping

12965 lncRNA Geno Rev (SALK_086573)	TTCCGCGGGATTAACTGTTAAAAGATTCAAAAAC	59.6	T-DNA insertion line genotyping
AtPSK3 LP (SALK_044781)	ATGTGTTACGCAGTTTCGTCC	55.6	T-DNA insertion line genotyping
AtPSK3 RP (SALK_044781)	AGCTTTGCTTCATGTTCTTGG	53.9	T-DNA insertion line genotyping
ACS6 Geno Fwd (SALK_054467)	AAAGATCTATGGTGGCTTTTGCAACAG	58	T-DNA insertion line genotyping
ACS6 Geno Rev (SALK_054467)	TTCTTAAGTTAAGTCTGTGCACGGACTAG	57.9	T-DNA insertion line genotyping
TST18 Geno Fwd (CS867285)	AAAGATCTATGTCTCAATCAATCTCCTCC	56.1	T-DNA insertion line genotyping
TST18 Geno Rev (CS867285)	TTCTTAAGTTAATTAGCAGATGGCTCCTC	56.5	T-DNA insertion line genotyping
PR5 LP (SALK_055063C)	CATTTCATTAATGGCTCGCTC	52.1	T-DNA insertion line genotyping
PR5 RP (SALK_055063C)	ATTGCTGTTATGGCCACAGAC	55.7	T-DNA insertion line genotyping
AGP14 LP (SALK_096806)	TTTAGGAGTTGTGCCCATGTC	55.1	T-DNA insertion line genotyping
AGP14 RP (SALK_096806)	CCTTAACGTGTCATAAATCAATTCC	52.4	T-DNA insertion line genotyping
tasi4 LP (SALK_066997)	CGAGGTTAAAATTCCGAAAGG	51.7	T-DNA insertion line genotyping
tasi4 RP (SALK_066997)	GTCCGCAATACGTAAAACTCG	54	T-DNA insertion line genotyping
miR163 LP Geno (CS879797)	ACCCGGTGGATAAAATCGAGTTC	57	T-DNA insertion line genotyping
miR163 RP (CS879797)	TCAAGCGTCCAGACTTCAGATTG	57	T-DNA insertion line genotyping
SAMP LP (SALK_209995C)	TGTTGCATTTGTGGACAAGAC	54	T-DNA insertion line genotyping
SAMP RP (SALK_209995C)	TGGAGTGATCTCGTAACGGAC	56.1	T-DNA insertion line genotyping
TAS3 RP2 (N432182 GABI-Kat)	TGAGAAGAGAGCAAAGAAACTTC	52.9	T-DNA insertion line genotyping
TAS3 LP2 (N432182 GABI-Kat)	CATGTGGAAACAAACGTATGAAG	52.6	T-DNA insertion line genotyping
GABI-Kat T-DNA primer 8474	ATAATAACGCTGCGGACATCTACATTTT	56.9	T-DNA insertion line genotyping
EXL1 Geno Fwd (SALK_010243C)	TCTATTACATTCGCGGCAATATTCG	55.4	T-DNA insertion line genotyping

EXL1 Geno Rev (SALK_010243C)	GCTATACGTGTAGGGCTCATAAGAC	56.5	T-DNA insertion line genotyping
MEE39 Geno Fwd (SALK_065070C)	ATGAAGAATCTTTGTTGGGTTTTTCTGTC	56.4	T-DNA insertion line genotyping
MEE39 Geno Rev (SALK_065070C)	GAACGATCATAAACATCTTTCGGGTAC	55.8	T-DNA insertion line genotyping
RBC3B Geno Fwd (SALK_117835)	AAAGATCTATGGCTTCCTCTATGCTCTCCTCCGC	64.3	T-DNA insertion line genotyping
RBC3B Geno Rev (SALK_117835)	TTGGTACCAAGAAATTAAGCTTCGGTGAAGCTTGG GG	65	T-DNA insertion line genotyping
ABAH3 Geno Fwd (SALK_078170)	AAGAGCTCATGGATTTCTCCGGTTTG	59.1	T-DNA insertion line genotyping
ABAH3 Geno Rev (SALK_078170)	TTGGTACCCTATGGTTTTCGTTCCAAGG	60.4	T-DNA insertion line genotyping
NRT2.6 LP (SALK_204101C)	CACCAAAGAGAGCTCCACAAG	55.7	T-DNA insertion line genotyping
NRT2.6 RP (SALK_204101C)	GGCTCTATTGGAACCTCCTTG	55.2	T-DNA insertion line genotyping
CUP LP (SALK_201444C)	CATCGTCACCACAATCTTTCC	53.9	T-DNA insertion line genotyping
CUP RP (SALK_201444C)	GGACAAAAGTTTGCATATGGC	52.8	T-DNA insertion line genotyping
AtNTR2.1 Geno Fwd (SALK_035429C)	GTTGGTTGCACATCATCATGGGAATCTTG	60.1	T-DNA insertion line genotyping
AtNTR2.1 qPCR Rev (SALK_035429C)	GGCGTCCACCCTCTGACTTG	60.4	T-DNA insertion line genotyping
OEP6 Geno Fwd (CS862774)	AAAGATCTATGGTGGAGAAGTCAGGAG	57.5	T-DNA insertion line genotyping
OEP6 Geno Rev (CS862774)	TCCTTAAGATTCTCACTCACCATATTCAGG	57.6	T-DNA insertion line genotyping
ESM1 LP (SALK_150833C)	TGAACGTCTGTGAAGTTCACG	55.2	T-DNA insertion line genotyping
ESM1 RP (SALK_150833C)	TGCCGGTTTTGTATTCTTGTC	53.6	T-DNA insertion line genotyping
RLD17 LP (SALK_115776C)	CAAGAGCTGAAAGCCTCAAAC	54.3	T-DNA insertion line genotyping
RLD17 RP (SALK_115776C)	TTACCAGGATGAGATGATCGG	53.7	T-DNA insertion line genotyping
PP2C LP (SALK_104445)	CACCAATCTTCATGGAGATCG	58.7	T-DNA insertion line genotyping
PP2C RP (SALK_104445)	GATTAATTTCGGCCAATGCTC	52.4	T-DNA insertion line genotyping

ADH1 LP (SALK_052699)	CGATGGGTACACCGATTACTG	55.1	T-DNA insertion line genotyping
ADH1 RP (SALK_052699)	AAAGATCGGCAACACATGATC	53.4	T-DNA insertion line genotyping
PERCB/34 (At3G49120) -OE-EcoRI-Fwd	AAGAATTCATGCATTTCTCTTCGTCTTC	55.9	Cloning of overexpression lines
PERCB/34 (At3G49120) -OE-BamHI-Rev	AAGGATCCTCACATAGAGCTAACAAAGTC	57.8	Cloning of overexpression lines
AGP31 (At1G28290) -OE-BglII-Fwd	AAAGATCTATGGGTTTCATTGGTAAGAG	55	Cloning of overexpression lines
AGP31 (At1G28290) -OE-BamHI-Rev	AAGGATCCTCATTTGGGGCAAGAC	59.3	Cloning of overexpression lines
HDT1/HDA3 (At3G44750) -OE-EcoRI-Fwd	AAGAATTCATGGAGTTCTGGGGAATTG	57.3	Cloning of overexpression lines
HDT1/HDA3 (At3G44750) -OE-BamHI-Rev	AAGGATCCTCACTTGGCAGCAGC	61.7	Cloning of overexpression lines
HDT3/HD2C (At5G03740) -OE-BglII-Fwd	AAAGATCTATGGAGTTCTGGGGTG	56.2	Cloning of overexpression lines
HDT3/HD2C (At5G03740) -OE-BamHI-Rev	AAGGATCCTCAAGCAGCTGCACTG	61.4	Cloning of overexpression lines
ROC2 (At3G56070) -OE-EcoRI-Fwd	AAGAATTCATGGCGAATCCTAAAGTC	55.5	Cloning of overexpression lines
ROC2 (At3G56070) -OE-BamHI-Rev	AAGGATCCTTATGAACTTGGGTTCTTGAG	58.3	Cloning of overexpression lines
